# From actin waves to mechanism and back: How theory aids biological understanding

**DOI:** 10.7554/eLife.87181

**Published:** 2023-07-10

**Authors:** Carsten Beta, Leah Edelstein-Keshet, Nir Gov, Arik Yochelis

**Affiliations:** 1 Institute of Physics and Astronomy, University of Potsdam Potsdam Germany; 2 Department of Mathematics, University of British Columbia Vancouver, BC Canada; 3 Department of Chemical and Biological Physics, Weizmann Institute of Science Rehovot Israel; 4 Swiss Institute for Dryland Environmental and Energy Research, Blaustein Institutes for Desert Research, Ben-Gurion University of the Negev, Sede Boqer Campus Midreshet Ben-Gurion Israel; 5 Department of Physics, Ben-Gurion University of the Negev Be’er Sheva Israel; Institut de Biologie du Développement France; Utrecht University Netherlands

**Keywords:** actin, waves, pattern formation, bifurcation, systems modeling, chemical signaling, Eukaryotic cells

## Abstract

Actin dynamics in cell motility, division, and phagocytosis is regulated by complex factors with multiple feedback loops, often leading to emergent dynamic patterns in the form of propagating waves of actin polymerization activity that are poorly understood. Many in the actin wave community have attempted to discern the underlying mechanisms using experiments and/or mathematical models and theory. Here, we survey methods and hypotheses for actin waves based on signaling networks, mechano-chemical effects, and transport characteristics, with examples drawn from *Dictyostelium discoideum*, human neutrophils, *Caenorhabditis elegans*, and *Xenopus laevis* oocytes. While experimentalists focus on the details of molecular components, theorists pose a central question of universality: Are there generic, model-independent, underlying principles, or just boundless cell-specific details? We argue that mathematical methods are equally important for understanding the emergence, evolution, and persistence of actin waves and conclude with a few challenges for future studies.

## Introduction

Unlike inanimate physical and chemical media, living systems feature a particular degree of complexity, hierarchical organization, and evolutionary structure. This poses unique challenges to the way in which we rationalize and systematically phrase our understanding of these systems, that is, how we set up models (in the broadest sense) of these complex living systems. Ideally, we would like such models to represent mechanistic reasoning (explanation) of the systems in a way that allows us to experimentally test predictions about the system’s behavior. For many biological questions, qualitative flowchart-type descriptions suffice (if gene X is knocked out, protein Y is not available any longer, which has this or that effect). However, in many situations, observations cannot be addressed by verbal arguments or cartoon-type approaches alone, and then, mathematical models are needed (Baker et al., 2008; Segel and Edelstein-Keshet, 2013; Ganusov, 2016; Fang et al., 2019). This applies not only to most quantitative questions but also to many qualitative features because typically the responses of biological systems to external changes or perturbations do not occur proportionally (linearly) but rather exhibit a more complex (nonlinear) dependence that is often unexpected.

Prominent examples are collective effects, where ordered large-scale patterns result from interactions and correlations of large numbers of molecular or nano-scale components of the system. The key characteristics of such structures are emergent properties in the sense that they do not reflect the properties of the individual building blocks in a linear, additive sense, but they are genuine ensemble properties that arise from the collective interactions of many constituents, that is, they only exist when large numbers of building blocks come together (Murray, 2001; Baker et al., 2008; Champneys et al., 2021). This may be seen in multicellular systems but also on the subcellular level (Edelstein-Keshet, 2005; Alon, 2006; Keener and Sneyd, 2009; Beta and Kruse, 2017; Halatek et al., 2018). Paradigmatic examples that have attracted much attention over the past decade are intracellular patterns that can be observed in the actin cytoskeleton of eukaryotic cells. From the collective action of large numbers of polymerizing actin filaments, motor proteins, and regulatory signaling components (Machesky and Insall, 1999), different macro-scale functional structures emerge that drive essential processes of cellular life (Kabsch and Vandekerckhove, 1992), such as motility, division, and nutrient uptake. Among them, wave patterns in the cell cortex have been observed as a common motif across many different cell types, such as human neutrophils, *Xenopus laevis* oocytes, and *Dictyostelium discoideum*. They have been intensely studied and became the target of many modeling efforts (Carlsson, 2010; Allard and Mogilner, 2013; Inagaki and Katsuno, 2017; Beta et al., 2020; Michaud et al., 2021; Povarova et al., 2023). Nevertheless, key questions remain open. For example, it is unclear to what extent the underlying mechanisms and the biological functions of actin waves are similar across different cell types, which of the wave characteristics are essential, and which are merely side effects of other, more essential, processes.

These questions pose many challenges to experimental research that can only be addressed together with theoretical methodologies. Due to the inherent complexity, models of actin waves typically lean on a reductionist approach (Carlsson, 2010), where the key players and their interactions are determined and cast into equations that recover the essential phenomena. However, as for many biological systems, it is not straightforward to identify a reduced model of actin waves. Their dynamics rely on dozens of molecular players (Kabsch and Vandekerckhove, 1992; Dominguez and Holmes, 2011), connected in a complex interaction network that is only partially known, it includes both chemical and mechanical factors, and features multiple feedback loops. Identifying the core molecular components that are essential for the formation of actin waves is a major outstanding challenge. Consequently, it often remains uncertain whether models of actin waves correctly represent the underlying mechanism, even if numerical simulations of the model reproduce the experimental behaviors.

Here, mathematics and, in particular, bifurcation theory can provide useful means to design and validate models, especially after simplifications have been introduced. Similar to the laws of thermodynamics which all physical systems have to obey, bifurcation theory imposes constraints on the form of a dynamic system that exhibits specific qualitative properties, such as excitability or oscillations, and explains their emergence, evolution, and persistence. It defines the different options and minimal mathematical features required to recover these properties, independent of the specific details of the model. The most notable example is the simplification of the Hodgkin–Huxley model of neuronal excitation to the phenomenological FitzHugh–Nagumo model (Ermentrout and Terman, 2010). Hodgkin and Huxley, 1952 proposed a seminal four-variable model based on voltage-gated ion channels to describe the propagation of an action potential along the membrane. But while the model captures realistic kinetics, the analysis and, thus, understanding its robustness is difficult. Instead, our understanding of many of the qualitative dynamic properties has been advanced through the analysis of the two-variable FitzHugh–Nagumo model (Fitzhugh, 1961; Nagumo et al., 1962), where one of the variables behaves as a switch (or excitable element) activating a second variable that damps the excitation via a slow negative feedback. While oversimplified from a physiological standpoint, the FitzHugh–Nagumo model is amenable to analysis and has proven valuable to understand the basic mechanisms of excitable and oscillatory dynamics, in general (Tyson and Keener, 1988; Meron, 1992). For that reason, models such as the FitzHugh–Nagumo model are frequently denoted as ‘toy models.’ A theoretical approach relying on reduced models is important for mapping out the range of possible behaviors, and for elucidating the dependence of simulated patterns on the chosen assumptions, details, and parameter regimes of a given model; in other words, for obtaining reliable conclusions and predictions that go beyond model-specific statements (Cross and Hohenberg, 1993; Champneys et al., 2021). It allows us to uncover model-independent ‘anchors’ around which specific model descriptions can be developed and, as such, provide important quality control and guideline for a reductionist model design.

In this review, we focus on actin waves to exemplify this approach. An analogy to electrophysiological waves in neurons and cardiac cells suggests that universal principles may also be inherent in self-organized cortical actin waves (Karma, 2013; Alonso et al., 2016). In addition, an increasing body of experimental results became available in recent years, which has prompted different modeling groups to focus on different specific experimental observations. We first summarize the current state of experimental research on intracellular actin wave patterns and then review the different modeling approaches that have been proposed to describe these phenomena. At the end, we provide a brief, non-technical introduction to the ideas of bifurcation theory and illustrate how these mathematical tools may be beneficial for guiding future modeling in this field.

## Experimental observations of actin waves

**Figure 1.**
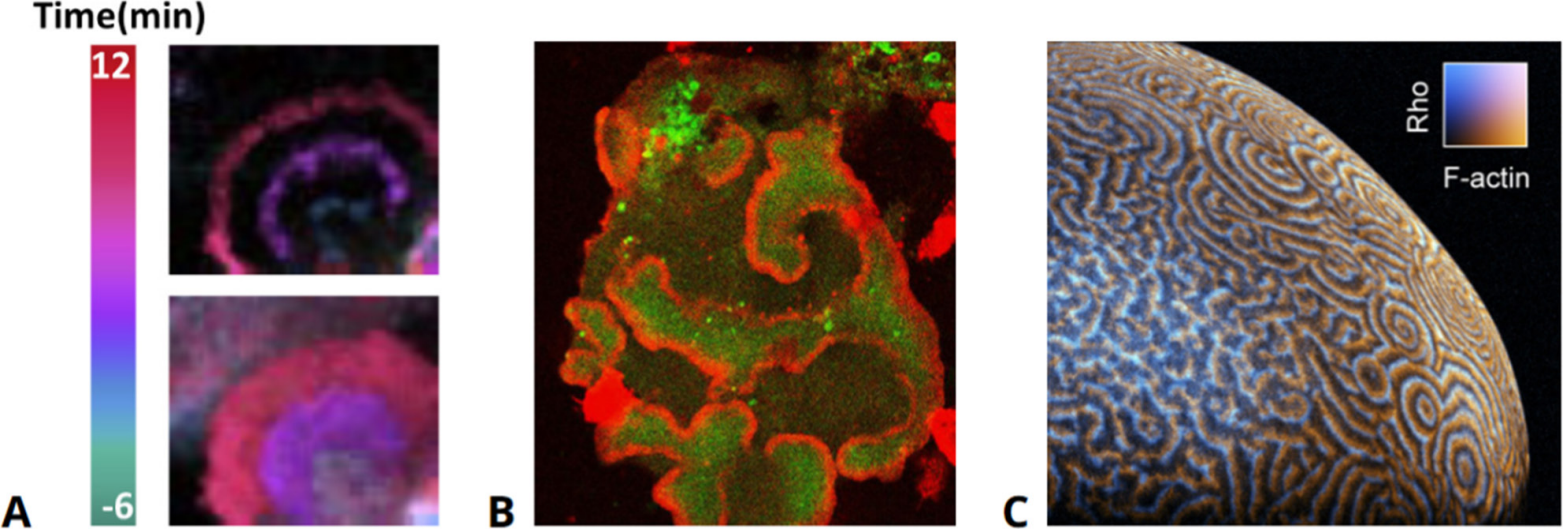
Examples of actin waves in various cell types. (**A**) Human epithelial cells exhibiting actin waves (top) associated with increased levels of PIP_3_ (bottom). (**B**) Actin waves (red) enclosing areas of elevated PIP_3_ levels (green) in an oversized *D. discoideum* cell, taken from data presented in Gerhardt et al., 2014a. (**C**) Waves of actin and Rho in the cortex of an immature *Xenopus* oocyte, modified from Michaud et al., 2022.

Actin waves are micron-scale cytoskeletal regions of increased filamentous actin density that propagate in a wave-like fashion in the actin cortex (Carlsson, 2010). They are typically also enriched in other actin-related proteins and regulatory components and may be associated with localized signaling processes at the adjacent plasma membrane. Actin waves have been observed in many different cell types, including human neutrophils (Weiner et al., 2007), dendritic cells (Stankevicins et al., 2020), neurons (Winans et al., 2016), T cells (Lam Hui et al., 2014), and breast cancer cells (Marchesin et al., 2015; Zhan et al., 2020). Besides examples from *Caenorhabditis elegans* (Michaux et al., 2018), actin waves have been particularly well studied in *D. discoideum* (Vicker et al., 1997; Gerisch et al., 2004; Arai et al., 2010) and *X. laevis* oocytes (Bement et al., 2015; Michaud et al., 2021; see also Figure 1 for examples from various species). Their size, morphology, speed of propagation, and biochemical composition may vary between different cell types. Yet, despite these diverse appearances, it is not clear how many truly distinct mechanisms give rise to actin waves in various cell types, nor how many variations of each underlying mechanism exist. The emergence of actin waves has been related to different cellular functions, such as motility, division, and phagocytosis (Carlsson, 2010; Inagaki and Katsuno, 2017). But also here, functional roles may vary between cell types. In some cases, it is unclear whether the wave patterns necessarily have a specific function or whether the waves are by-products of signaling dynamics or ever-changing cell morphology.

Wave-like dynamics of actin structures were reported already several decades ago, starting with circular dorsal ruffles (CDRs) in the 1970s (Abercrombie et al., 1970) in various cell types, such as fibroblasts and glial cells, in response to receptor-tyrosine-kinase growth factors (Buccione et al., 2004). CDRs are transient actin-based structures that consist of a dynamic ring-shaped ridge expanding and contracting across the dorsal plasma membrane in a meandering, wave-like fashion (Chhabra and Higgs, 2007; Swanson, 2008). A different but potentially related wave phenomenon was observed in the mid-1990s, where protrusions of the cell border were reported that move along the periphery of *D. discoideum* cells and human epidermal keratinocytes (Killich et al., 1993; Alt et al., 1995). With the rapid improvements in live-cell fluorescence microscopy, these findings were soon complemented with real-time observations of wave dynamics at the cytoskeletal level relying on fluorescent markers of actin and actin-related proteins in *D. discoideum* (Vicker et al., 1997; Vicker, 2002b; Gerisch et al., 2004; Bretschneider et al., 2004) and in other cell types (Vicker, 2002a; Weiner et al., 2007). They triggered growing interest in the phenomenon of actin waves, resulting in a boost of experimental and theoretical studies over the following decades.

A strong focus of the earlier work in this field was on periodic and wave-like morphodynamics of the cell outline (Verkhovsky, 2015). In spreading mouse embryonic fibroblasts, periodic contractions of lamellipodia were found to correlate with rearward moving actin waves (Giannone et al., 2004; Giannone et al., 2007). Lamellipodial protrusions and retractions may also travel laterally along the membrane (Döbereiner et al., 2006; Machacek and Danuser, 2006), as observed in fish epithelial keratocytes (Barnhart et al., 2011; Lou et al., 2015; Barnhart et al., 2017), *D. discoideum* (Driscoll et al., 2012; Driscoll et al., 2015), and *Xenopus* carcass fibroblasts (Ryan et al., 2012a). Besides wave dynamics at the leading edge of spreading and migrating cells, wave-like structures were also reported from cultured neurons that may exhibit actin-based, fin-like membrane protrusions traveling along their thin axonal extensions (Ruthel and Banker, 1998; Toriyama et al., 2006; Tomba et al., 2017). However, fin-like actin waves are not restricted to neural cells but may also occur in other cell types when cultured on thin fibers (Guetta-Terrier et al., 2015).

Wave dynamics is not only observed at the cell border. Traveling actin waves also emerge at the ventral and dorsal cell surfaces. For example, in human neutrophils, propagating waves of Hem-1, a member of the WAVE complex that regulates Arp2/3 activity, together with actin assembly were observed (Weiner et al., 2007; Millius et al., 2009). These waves show signatures of excitable behavior and organize the protrusion dynamics at the leading edge of migrating neutrophils. Similarly, the amoeboid motion of dendritic cells that mediate immune responses to various pathogens is affected by the presence of actin waves that can induce switches between diffusive and persistent states of motion (Stankevicins et al., 2020). Ventral actin waves can also associate with integrin-mediated adhesions to form so-called adhesive F-actin waves, as was observed in several mammalian cell lines (Case and Waterman, 2011). However, in many cases it is not clear whether the waves are generated at the cytoskeletal level or merely reflect wave dynamics that emerges in the upstream signaling network (see Box 1 for an overview of some of the major signaling components regulating actin assembly). In rat basophilic leukemia mast cells, for example, calcium oscillations may change the dynamic character of the actin wave patterns (Wu et al., 2013) and distinct phosphoinositides control the frequency and amplitude of cortical oscillations (Xiong et al., 2016). In oocytes and embryonic cells of frogs and echinoderms, Rho signaling activation together with inactivation by the GAP RGA3/4 (a GTPase-activating protein recruited by F-actin) result in excitable waves (Bement et al., 2015) that were successfully reconstituted also in an in vitro system (Landino et al., 2021). Important functions during cell division, such as division plane placement, have been attributed to this form of ‘cortical excitability’ (Michaud et al., 2021) and to the onset of excitable behavior (Swider et al., 2022; Michaud et al., 2022).

Box 1. **Common regulators of actin assembly**

Box 1—figure 1 represents some of the most common regulators of actin polymerization (found in many eukaryotic cells) and a few of the feedback interactions identified experimentally in various species.

The assembly of filamentous actin (F-actin, branched structures) takes place close to the cell membrane. Membrane-bound complexes (WASP or WAVE, yellow ovals) activate a protein that forms the actin branch-points (Arp2/3, blue dots; Svitkina and Borisy, 1999; Blanchoin et al., 2000). Other membrane binding proteins such as formins (e.g., mDia, not shown) also lead to growth of F-actin strands. The actin filament tips (‘barbed ends’) exert force on the cell membrane (Mogilner and Oster, 2003), leading to protrusion of the cell edge. F-actin assembly is promoted by GTPases such as Rac and Cdc42 (which activate WASP, WAVE) and by Rho (which activates mDia; Watanabe et al., 1997).

In *D. discoideum* and neutrophils, branching nucleation dominates at the cell front (Glogauer et al., 2000; Welch and Mullins, 2002). Various regulators, including Hem-1 (a membrane-resident part of the WAVE2 complex) in neutrophils (Weiner et al., 2007), participate in feedback loops activating Arp2/3. In many mammalian cells, GTPases such as Rac, Cdc42, and Rho are central to cell polarity, motility, and F-actin dynamics (Nobes and Hall, 1995; Etienne-Manneville and Hall, 2002). In *D. discoideum*, the GTPase Ras plays a similar role (Kortholt and van Haastert, 2008). GTPases are molecular switches, with an active, membrane-bound ‘ON’ form (dark green octagon), and an inactive cytosolic ‘OFF’ form (light green). Only the active form has downstream action on other effectors. GTPases are activated by guanine nucleotide exchange factors (GEFs) and inactivated by GTPase-activating proteins (GAPs) (Hodge and Ridley, 2016). In some systems, including embryos of *C. elegans*, *Xenopus*, and starfish, F-actin is assembled by mDia, which is activated by Rho (Bement et al., 2015; Michaux et al., 2018; Michaud et al., 2021; Yao et al., 2022).

Phosphoinositides (green quadrilaterals) are membrane lipids involved in signaling to actin. PIP_3_, the triply phosphorylated version (dark green), greatly accelerates F-actin assembly and is correlated with Rac activity (Van Keymeulen et al., 2006). PIP_3_ is generated from its less active form, PIP_2_ (light green) by the kinase Phosphoinositide 3-kinase (PI3K, adds a phosphate group), and converted back to PIP_2_ by the Phosphatase and Tensin homolog (PTEN) (Kölsch et al., 2008). Since PI3K and PTEN spatially exclude each other, they may be mutually antagonistic (Jiang and Liu, 2008). PI3K and PTEN have active membrane-bound forms and inactive cytosolic forms, with rapid exchange rates (secs) that may be affected by feedback from PIP_2_ and PIP_3_ (Hilgemann, 2007). On the timescale of actin waves (seconds, minutes), the total amounts of PI3K, and of a given GTPase is roughly constant, since rates of synthesis or degradation of such proteins are much slower (hours) (Spiering and Hodgson, 2011), justifying a common modeling assumption of conservation.

**Box 1—figure 1.**
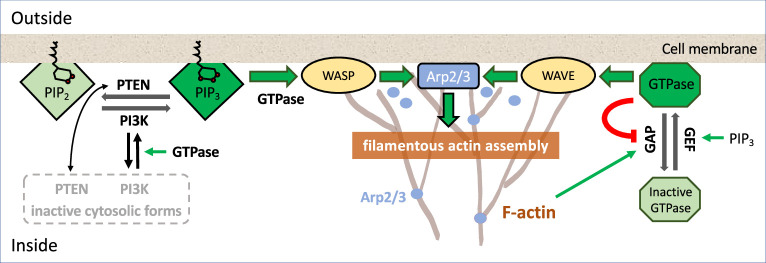
An example of signaling pathways. Schematic diagram of a partial set of key players signaling to F-actin assembly; colored lines represent interconversion (black), positive feedback (green), negative feedback (red), and dark (light) shades of similar background/text colors denote active (inactive) forms. For more detailed biochemical pathways, see, for example, Devreotes et al., 2017; Avila Ponce de León et al., 2021.

It is accepted that myosin also plays a role in excitability, contractility of the cell cortex, and/or oscillatory behavior. For example, cycles of periodic lamellipodial contraction critically depend on myosin II activity (Giannone et al., 2007). Furthermore, the works of Michaud et al., 2021 and those leading up to it implicitly connect Rho signaling to myosin contractility, though in many studies myosin is lumped with F-actin in what is denoted as ‘actomyosin’ (e.g., see Driscoll et al., 2015). More explicit experimental probing of the spatial waves of Rho and myosin in cells is shown in Graessl et al., 2017. The role of feedback from myosin back to its signaling regulators has also been explored in cells confined to 1D by Hadjitheodorou et al., 2021. Here, the behavior is reduced to front-back cell repolarization under specific conditions (a geometrically simple form of a ‘1D actomyosin wave’). While important, in this review we limit our main focus to ‘actin waves,’ and only occasionally mention myosin as a separate player.

One of the most thoroughly studied examples of ventral actin waves are those observed in *D. discoideum* (Vicker, 2002b; Bretschneider et al., 2004; Gerisch et al., 2004). These waves are composed of large ring-shaped actin structures that deform and meander across the substrate-attached bottom membrane and extend 1–2 µm into the cytosolic region (Bretschneider et al., 2009). The F-actin ring separates an inner region enriched in F-actin and Arp2/3 from the outer part of the cortex characterized by increased levels of cortexilin I, myosin II, and different formins (Schroth-Diez et al., 2009; Ecke et al., 2020). In addition, both regions exhibit distinct network architectures (Jasnin et al., 2019) and surface charges (Banerjee et al., 2022). The different class I myosins colocalize with the wave or the inner region and may inhibit wave formation in a lipid-binding dependent manner (Brzeska et al., 2014; Brzeska et al., 2020). When traveling over larger cortical areas of oversized cells, waves maintain a preferred size and may annihilate upon collision (Gerhardt et al., 2014a; Miao et al., 2019). This cytoskeletal organization is coupled to wave patterns in the upstream signaling system, such that the inner patch enclosed by the actin wave is enriched in activated Ras and PIP_3_, whereas PTEN and PIP_2_ are found outside the ring (Asano et al., 2008; Gerisch et al., 2011). A rich variety of dynamic modes was observed for these patches, including alternating oscillations, rotational movement, and periodic switching between the ventral and dorsal membranes (Gerisch et al., 2011; Gerisch et al., 2012; Helenius et al., 2018). Many of these dynamic patterns are preserved in the signaling systems even if cytoskeletal activity is suppressed by treatment with inhibitors of actin polymerization (Arai et al., 2010; Fukushima et al., 2019; Hörning and Shibata, 2019).

Actin waves in *D. discoideum* have been associated with various cellular functions. When colliding with the cell border, they push the membrane outward (Vicker, 2000; Bretschneider et al., 2009; Taniguchi et al., 2013). Even though not strictly required for cell motility, waves may also affect the movement by triggering transitions between the amoeboid and a more persistent, keratocyte-like mode of locomotion (so-called fan-shaped cells) (Asano et al., 2008; Miao et al., 2017; Cao et al., 2019b; Moreno et al., 2020; Ghabache et al., 2021; Moldenhawer et al., 2022). Moreover, waves may also drive the division of cells (Flemming et al., 2020). While initiation and guidance of waves by chemoattractants was not observed (Gerhardt et al., 2014b; Lange et al., 2016), electrical fields affect the wave propagation and result in more abundant formation of protrusions in the field’s direction (Yang et al., 2022). Similarly, nano- and micrometer-sized surface ridges guide the propagation of actin waves (Driscoll et al., 2014; Sun et al., 2015; Honda et al., 2021). The ring-shaped actin waves in *Dictyostelium* furthermore show structural similarities with phagocytic cups (Gerisch et al., 2009; Gerisch, 2010), an observation that has also been reported for macrophages (Masters et al., 2016). These waves may thus serve as precursors of macropinocytic cups (Veltman et al., 2016; Lutton et al., 2022), which is also supported by the vertical orientation of the actin filaments inside the ventral ring-shaped structures, indicating a force generating machinery that may drive the formation of an outward protruding cup (Jasnin et al., 2019).

## Mathematical modeling approaches

The broad range of actin wave patterns has stimulated a wide spectrum of modeling approaches, varying greatly in their complexity and level of detail (Ryan et al., 2012b; Allard and Mogilner, 2013; Sept and Carlsson, 2014; Beta and Kruse, 2017; Beta et al., 2020). Some works start with detailed signaling circuits of interacting components (Khamviwath et al., 2013; Avila Ponce de León et al., 2021), resulting in models that consist of large sets of coupled equations. In most situations, the exact functional forms and parameter values cannot be uniquely determined from experimental observations and are somewhat arbitrary. The analysis of such models mainly relies on numerical simulations (numerical integration of the model equations), often yielding results that quantitatively match specific experimental data after adjusting parameters. However, it is not always clear how robust these findings are with respect to model details and whether the agreement with the experimental data provides valid intuition about the underlying mechanism.

Along with these detailed approaches, there are many attempts to formulate models that reduce the complexity by focusing on a specific part of the signaling or cytoskeletal system. These models contain a lower number of equations, while still maintaining an explicit biological interpretation of the model variables; see, for example, Table 1. Identifying the core wave mechanisms in a given circuit implies that layers of complexity are removed to find the essential underlying motif that accounts for the observed patterns. Here, the role of actin in the generation of waves is a central aspect to link model predictions to experimental findings. Largely, this involves experimental observations of waves in systems where mutations, knockouts, or drug treatments are used to eliminate specific components of the signaling circuit. We will provide examples of this approach in the context of *D. discoideum* below. However, whether actin plays similar roles for wave generation in different cell types is an open question. Note also that some of the reduced models are even more simplified. They are designed to capture the observed macroscopic patterns with the most minimal mathematical ingredients. Here, the choice of model equations is mainly driven by mathematical intuition and only to a lesser extent by biological mechanisms and signaling motifs. Consequently, the model variables only maintain a vague biological interpretation and are mostly seen as effective, lumped quantities that represent the joint action of many involved players.

**Figure 2.**
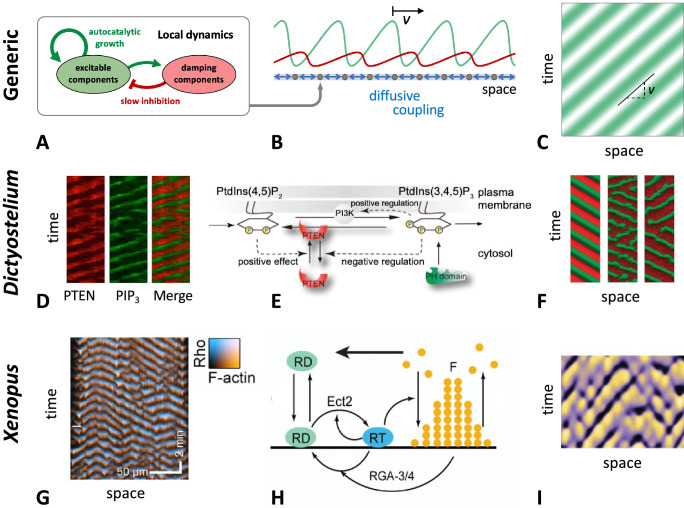
General principle of actin wave formation. Most actin wave models are based on local nonlinear processes that involve positive and negative feedbacks between interacting species (**A**). In an extended system with spatial coupling, such as diffusive transport (**B**), this may give rise to propagating waves (**C**), where v is the wave propagation (group) speed. Examples of actin waves and models in *D. discoideum* (**D–F**) and *Xenopus* oocytes (**G–I**) showing kymographs of the experimentally observed waves (**D, G**), model schematics (**E, H**), and simulations (**F, I**). The models proposed by Arai et al., 2010; Shibata et al., 2012; Shibata et al., 2013 consider active PTEN, PIP_2_, and PIP_3_, and assume conservation of PTEN. The models in Goryachev et al., 2016; Michaud et al., 2022 are based on Rho (RD, RT) and its GAP (RGA-3/4) interacting with F-actin (**F**). (**D–F**) were modified from Arai et al., 2010, Figures 1D, 5A and D, respectively. (**G–I**) were modified from Michaud et al., 2022, Figures 2C, 7A and E, respectively.

In this section, we will summarize the large body of work on actin wave models that are of reduced complexity in the sense explained above. In general, they all rely on a set of interacting species that are connected via positive and negative feedback loops, resulting in a nonlinear temporal dynamics at each location in the cell cortex (e.g., switch-like or time-periodic behavior). In addition, the local dynamics is spatially coupled to adjacent locations in the cortex, for example, via diffusive transport of some of the involved components or as a consequence of nonlocal changes in the mechanical properties. Local changes will thus affect the dynamics at neighboring points in the cortex, which may result in the formation of propagating waves, see Figure 2A–C for a schematic representation of this generic principle. Mathematically speaking, most of these models belong to the so-called reaction-diffusion-type, where spatial coupling is established by diffusive transport. However, extensions and alternative approaches will be also mentioned at the end of this section. Note, however, that we focus on descriptions of actin waves under spatially isotropic, homogeneous conditions and do not elaborate on the impact of externally applied chemical gradients, electric fields, or other directional cues.

**Table 1.** Representative mathematical models with their main variables, additional components, and methods of study.

Species	Model	NPF	F	I	PI	Other components	Study methods	Reference
*Dictyostelium*	RD				✔	PIP_2_, PIP_3_, PTEN	SIM, linear BIF	Shibata et al., 2012
*Dictyostelium*	RD	✔	✔	✔	✔	Arp2/3, Coronin, Rac, WASP, etc.	SIM	Khamviwath et al., 2013
*Dictyostelium*	RD, PF				✔	PIP_2_, PIP_3_, PTEN, PI3K	SIM, linear BIF	Taniguchi et al., 2013
*Dictyostelium*	RD		✔	✔		Monomers, Coronin	SIM	Wasnik and Mukhopadhyay, 2014
*Dictyostelium*	SRD, PF				✔	PIP_3_, PTEN	SIM	Knoch et al., 2014
*Dictyostelium*	RD				✔	Ras, PTEN, GAP, PIP_3_	SIM	Fukushima et al., 2019
*Dictyostelium*	SRD, LSM		✔	✔	✔	PIP_2_, Ras/Rap, PKBs, Rac, Coronin	SIM	Miao et al., 2019
*Dictyostelium*	SRD, PF			✔	✔	Cell edge, cytofission	SIM	Flemming et al., 2020
Echinoderm	RD	✔	✔			Rho, Ect2	SIM	Bement et al., 2015
*Xenopus*	RD	✔	✔			Rho, Ect2	SIM	Michaud et al., 2022; Michaud et al., 2021
Cell extracts	RD	✔	✔			Rho, Ect2	SIM	Landino et al., 2021
*C. elegans*	ODE	✔				Rho, RGA3/4	SIM	Michaux et al., 2018
Neutrophil	ABM, ODE	✔	✔			Hem1	SIM	Weiner et al., 2007
Fibroblast	RD		✔	✔		Cortical actin/stress fibers, cell edge	SIM, linear BIF	Bernitt et al., 2017
General	SRD		✔	✔		F-actin orientation	SIM	Whitelam et al., 2009
General	ABM	✔	✔			Filament network	SIM	Carlsson, 2010
General	RD	✔	✔			Elasticity, cell edge	SIM	Doubrovinski and Kruse, 2011
General	RD	✔	✔	✔		GTPases for nucleation	SIM, LPA	Holmes et al., 2012
General	SRD, PF		✔			Cell edge, cell-to-cell variability	SIM	Alonso et al., 2018
General	RD		✔	✔		G-actin	SIM, nonlinear BIF	Yochelis et al., 2022

NPF, actin nucleation promoting factor; F, F-actin; I, inhibitor; PI, phosphoinositides; SIM, simulations via numerical integration; BIF, bifurcation analysis; ABM, agent-based model; ODE, ordinary differential equations; RD, reaction-diffusion system; PF, phase field equations; SRD, stochastic-reaction-diffusion system; LPA, local perturbation analysis; LSM, level set method.

### Reaction-diffusion-type models

Most actin wave models describe the interactions of molecular players using traditional chemical reaction kinetics localized at the cell cortex or/and the membrane (local dynamics, see Figure 2A). In addition, spatial coupling is assumed, typically based on diffusive transport of the species involved (see Figure 2B). Such models can be mathematically expressed as reaction-diffusion (RD) systems, composed of reaction terms that represent the local intracellular kinetics and a diffusion term, accounting for the diffusive transport of all or some of the species. The reaction terms involve nonlinear interactions between the reacting species that are often assumed to be of Michaelis–Menten type (saturating) or switch-like (sigmoidal, a.k.a. ‘Hill function’ type). While the number of interacting species can be large, it is often possible, under certain assumptions, to reduce the number of variables in an RD model to three, two, or even a single dynamic variable in the case of minimal phenomenological models, as briefly discussed in Box 2. Due to the large body of such works, here we survey them according to different cell types (species).

Box 2. **Methods of reduction for systems of differential equations**

The complexity of cellular systems tends to obscure the identities of elements that act as ‘central organizers.’ Mathematical methods can offer significant model simplification (reduction) in whittling away the peripheral vs. central components. In what follows, we highlight some common methods.

**Dimensional analysis**

Rescaling variables in terms of ‘baseline values’ (for concentrations, fluorescence levels, etc.), relevant timescales (reciprocals of typical decay rates), or typical lengthscales (e.g., cell diameter or diffusion lengthscale) can reduce the number of free parameters (Lin and Segel, 1988; Murray, 2002; Breña–Medina et al., 2014; Holmes et al., 2015; Bernitt et al., 2017; Verschueren and Champneys, 2017). In some cases, for example, Flyvbjerg et al., 1996; Bailey et al., 2011, this step, together with data that follows some ‘qualitative trends,’ can already reveal precise underlying molecular mechanisms that are unknown ab initio (i.e., from first principles).

**Scale separation analysis**

Rescaling (i.e., reducing a model to a dimensionless formulation as mentioned above) can reveal additional relative rates of underlying processes, such as rate-limiting steps for chemical reactions. Then, it is possible to make simplifying assumptions (replace some variables by ‘slowly changing parameters’) in a process denoted quasi steady-state analysis (QSS). This can also help to reduce a large and complicated model into a hierarchy of submodels operating at fast, medium, and slow timescales. Each can be analyzed in turn in a more complete way, for example, see Keener and Sneyd, 2009; Desroches et al., 2022; Tran et al., 2009; Newby and Bressloff, 2010; Plazen et al., 2023; Plazen and Khadra, 2023. Rescaling can also identify submodels or processes that are governed by a ‘small (dimensionless) parameter’ (e.g., ratios of rates of diffusion of the membrane-bound and cytosolic forms of regulatory proteins). Then, asymptotic analysis (a.k.a. singular perturbation theory) can help to decipher how system behavior depends on such quantities.

**Piecewise-linear approximations**

Many models for biochemical interactions exploit Hill functions to depict switching between states (e.g., self-amplified rates of activation). Approximating such terms by ‘sharp switches,’ that is, step functions or piecewise-linear terms (Gebert et al., 2007), can vastly simplify the process of solving for steady states explicitly (hence bypassing the need for numerical analysis). In some cases, for example, Holmes and Edelstein-Keshet, 2016; Holmes et al., 2016, this provides a reasonable first step in mapping out the parameter dependence of various regimes.

**Reducing PDEs to ODEs**

Approximating or rewriting PDEs by ODEs can also vastly increase the availability of methods of analysis. Local perturbation analysis (LPA) exploits the differences between slow and rapidly diffusing components, approximating these by zero and infinite rates of diffusion, thereby defining ‘local’ and ‘global’ variables. LPA can then be used in conjunction with (simpler) ODE tools to find parameter regimes in which a localized stimulus is expected to generate ‘interesting behavior,’ examples include Marée et al., 2006; Holmes et al., 2012; Holmes et al., 2015; Mata et al., 2013; Nakamura and Shibata, 2015. Another form of reduction is by transformation based on a conserved property, for example, for steady propagating patterns it is convenient to use Galilean transformation (x,t)→ξ=x±vt (a.k.a. a co-moving coordinate frame transformation), where v is a propagation speed. Examples in the context of actin waves include Yochelis et al., 2020; Champneys et al., 2021; Yochelis et al., 2022.

All the above methods contribute to the ability to analyze the model equations and facilitate the use of bifurcation theory to uncover the origin of wave mechanisms, as elaborated in the text.

#### Models of actin waves in *Dictyostelium*

Reduced mechanistic models of actin waves critically depend on identifying the core wave generator of the system. Here, we will present examples from *D. discoideum* to demonstrate how the list of key components can be narrowed down by combining experimental and modeling studies. In 2010, traveling waves of PIP_3_ and PTEN were reported from cells treated with Latrunculin A, a drug that inhibits actin polymerization (Arai et al., 2010), demonstrating that neither F-actin nor the chemoattractant cAMP are needed for wave generation (only later it was argued that F-actin could play a stabilizing role in wave dynamics; see Nishikawa et al., 2014). These observations inspired a model in which the key dynamics is maintained via PIP_2_/PIP_3_ signaling (see Figure 2D–F; Arai et al., 2010; Shibata et al., 2012). The model is based on assuming that PIP_3_ negatively regulates membrane association of PTEN, while PIP_2_ helps to activate PTEN, thus effectively providing a positive feedback from PTEN to itself. PI3K was taken to be independent of PIP_2_ and PIP_3_, and constant in the absence of chemoattractants. PTEN was assumed to be well-mixed in the cytosol and non-diffusive on the membrane originally, but diffusion was included in a later variant of the model (Shibata et al., 2013). Numerical simulations reproduced the experimentally observed signaling waves and some analysis was also performed (Nakamura and Shibata, 2015), including a ‘local perturbation analysis’ (LPA) (Holmes, 2014).

Subsequent experiments demonstrated that excitable behavior can also be present (Huang et al., 2013; Nishikawa et al., 2014). It was observed that Ras activation and increased levels of PI3K are associated with the wave patterns (Fukushima et al., 2019). Moreover, based on more recent fluorescence recordings, Ras appears as the central player of the wave generator, in that Ras waves affect PI3K and PIP_3_ but can persist independently of those intermediates (Fukushima et al., 2019). In light of these newer findings, an extended model was proposed that includes active and inactive forms of Ras, as well as an unspecified Ras GAP, while the kinetics of PIP_2_ and PIP_3_ are included in a simplified form (Fukushima et al., 2019). Important features of the model are that PIP_3_ helps to recruit Ras to the membrane in a positive feedback loop, and both the active and the inactive Ras affect the Ras GAP. Besides models that exclusively focus on a wave generator in the signaling system, wave dynamics in *D. discoideum* has also been addressed by so-called ‘excitable network models’ (Xiong et al., 2010; Pal et al., 2019). Here, two excitable modules are coupled together that represent the signaling and cytoskeletal dynamics (Huang et al., 2013), respectively. They have been successfully adapted to reproduce many experimental observations of actin wave patterns under a wide range of different conditions (Miao et al., 2017; Miao et al., 2019).

Reaction-diffusion models of intracellular waves in *D. discoideum* were also studied inside deforming model cells. The dynamic phase field approach represents cell shape by a function that takes on distinct values (e.g., 1 or 0) inside versus outside of the cell with a smooth interface connecting these values along the cell boundary (e.g., see Shao et al., 2010; Camley et al., 2017). This approach has been used, for example, in a study focusing on the dynamics of phase singularities in a variant of the PIP_2_/PIP_3_ model (Taniguchi et al., 2013). A phase field model for the dynamics of PIP_3_ and PTEN has also been extended by a noisy excitable module to account for the observations of transient ‘holes’ in the PTEN distribution (Knoch et al., 2014). In Ghabache et al., 2021, different distributions of actin and myosin were prescribed within the phase field domain to account for different motility modes. Finally, with further reduction, generic wave generators (a.k.a. ‘toy’ models) have been combined with a dynamic phase field to qualitatively address a wide range of experimental observations in *D. discoideum*. Even though the relation of these models to individual molecular players in the cell often remains vague, they successfully captured observations of cell-to-cell variability (Alonso et al., 2018), of different motility phenotypes (Cao et al., 2019b; Moreno et al., 2020; Moldenhawer et al., 2022), and of wave-mediated cell division events (Flemming et al., 2020). In combination with an F-actin orientation field, a noisy FitzHugh-Nagumo-type model also explains how actin spots become mobile and form traveling actin waves (Whitelam et al., 2009).

#### Models of actin waves in oocytes

In cell division, signals from the mitotic spindle set up waves of Rho and F-actin along the cortex, which are funneled to the cell’s equator, where the contractile ring eventually splits the mother cell into its two daughter cells. In immature oocytes of frogs (*X. laevis*) and starfish (*Patiria miniata*), where the cortex is quiescent, vibrant dynamic wave patterns of Rho and F-actin can be induced by expressing two regulators of Rho (the GEF Ect2 and the GAP RGA3/4) at various ratios (Michaud et al., 2021; Michaud et al., 2022). Here, waves of F-actin are observed to follow closely behind waves of activity of the GTPase Rho. A model of this system was proposed taking into account diffusively mobile active and inactive Rho, stationary F-actin, an autocatalytic coupling of Rho to itself via the Rho-GEF Ect2, as well as inactivation of Rho by F-actin, see Figure 2G–I (Goryachev et al., 2016; Michaud et al., 2021). Recent experiments have confirmed that the core circuit of F-actin, Ect2, and the Rho-GAP RGA-3/4, which was identified as the negative regulator correlated with F-actin feedback, controls the dynamics of patterns in the cortex of oocytes (Michaud et al., 2022). Based on extracts of *Xenopus* oocytes and artificial lipid membranes, the Rho and F-actin wave patterns could even be reconstituted in vitro (Landino et al., 2021). Traveling waves were observed that emerge from random foci and expand as target waves that annihilate upon collision. Surprisingly, even diluted extracts displayed similar robust dynamics. Inhibiting Rho or actin polymerization destroyed the dynamics, proving that both components are essential for the waves. In the abovementioned models of actin waves in *X. oocytes,* the role of myosin in cortical contraction is not modeled explicitly.

Disordered wave patterns in the starfish oocyte system have also been analyzed from the viewpoint of spiral wave turbulence (Tan et al., 2020). Comparing the experimental observations to the complex Ginzburg–Landau (CGL) equation, which describes the dynamics of any reaction-diffusion system close to the onset of oscillations, suggests that the observed spiral wave properties are generic (model independent) features. However, while this model is based on the creation/annihilation dynamics of topological defects (intrinsically arising dislocations in the phase field) at the cores of the Rho spiral vortices, it is only indirectly related to the specific underlying biological mechanisms.

#### Models of actin waves in various other species

**Figure 3.**
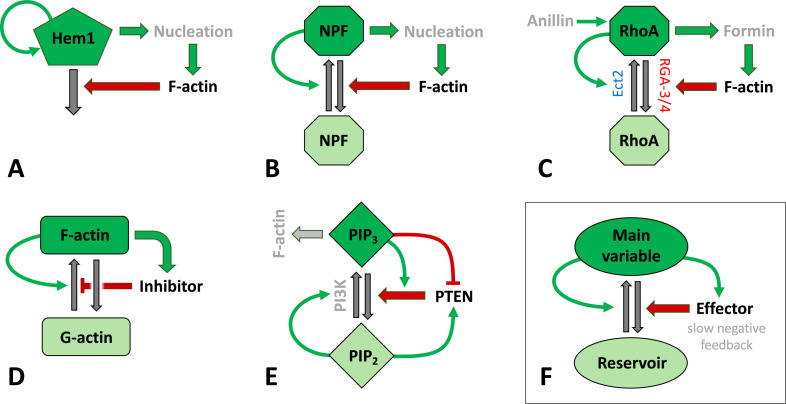
Schematic representation of signaling mechanisms for actin waves identified in various species and included in many models**.** Green (red) arrows correspond to positive (negative) feedback signaling (red arrows promote inactivation in **A–C, E–F** or inhibit actin assembly in **D**). Black arrows represent interconversions, and dark (light) colors of shapes correspond to active (inactive) forms of signaling agents. (**A**) The Hem1 complex was identified as a central component in neutrophils as modeled in Weiner et al., 2007. (**B**) A generic model for actin waves based on the interactions of F-actin with a nucleation promoting factor (NPF) (Doubrovinski and Kruse, 2011; Dreher et al., 2014; Holmes et al., 2012; Mata et al., 2013). A similar filament model by Carlsson, 2010 left out the autocatalysis and failed to produce well-structured waves. (**C**) The assembly of F-actin by the formin mDia was investigated in embryos of *C. elegans* (Michaux et al., 2018), where RGA3/4 was identified as the GAP. In oocytes of *Xenopus* and starfish, Ect2 was found to be the GEF, and RGA3/4 is recruited by F-actin (Bement et al., 2015; Goryachev et al., 2016; Michaud et al., 2021). The circuit was modeled in Goryachev et al., 2016. (**D**) A model for F- and G-actin and a polymerization inhibitor was proposed by Yochelis et al., 2020; Yochelis et al., 2022. There are also similar, yet more detailed versions, that identified coronin as the inhibitor (Wasnik and Mukhopadhyay, 2014) or that incorporated cortical actin/stress fibers (Bernitt et al., 2017). (**E**) A slightly distinct model structure for phosphoinositides and PTEN as proposed in Shibata et al., 2012. (**F**) Essential structure of models (**A–D**): The key variable is autocatalytic and promotes an effector that exerts at least one (slow) negative feedback. The circuits have been drawn to emphasize similarities in the structures and connectivities. In (**B–D**), the total amount of the main agent (RhoA, GTPase, or actin) was assumed to be constant.

In neutrophils, the dynamics of the Hem-1 component of the SCAR/WAVE complex gives rise to actin waves that correlate with cell edge protrusions (Weiner et al., 2007). As demonstrated in this pioneering work, Hem-1 can self-activate and is negatively regulated by F-actin (via an unknown mechanism), as shown in Figure 3A. Assuming a constant total pool of actin that polymerizes in a Hem-1-dependent fashion, a simple model that includes only Hem-1 and F-actin was proposed that produced waves resembling the experimental observations (Weiner et al., 2007). Rather than local coupling by diffusion (associated with nearest-neighbor interactions), the authors used a nonlocal kernel (integral convolution with a Gaussian) to model actin-dependent autoactivation of Hem-1 over some spatial neighborhood.

In early *C. elegans* embryos, control cells show oscillating focal pulses of Rho activity, rather than oscillatory waves (Michaux et al., 2018; Yao et al., 2022). The GTPase Rho leads to actin assembly by formins of the Dia family, a route that is parallel to the ‘common’ WAVE/WASP activation of Arp2/3-dependent actin branching. Here, negative feedback stems from the Rho GAP (RGA-3/4) that gets recruited by F-actin and locally inactivates Rho, as shown in Figure 3C. Recently, it was demonstrated that well-controlled perturbations to anillin or formin affect the nature of the observed patterns (see also Bolado-Carrancio et al., 2020 for a study that combines experiments on breast cancer cells and a model that explains propagating waves of Rac and Rho in cell migration). Rho is also known to activate myosin contraction, a feature that many of the above models do not address. One exception is the recent model of Staddon et al., 2022, where actomyosin leads to contraction and also creates stress that feeds back to Rho activation. This is, however, a time-dependent model with as yet no spatial degrees of freedom.

In a different approach, several models assign autocatalysis to F-actin itself, for example, mediated by Arp2/3 branching, and propose or identify an additional inhibitor of assembly or a promoter of disassembly, as shown in Figure 3D. A simple example of this type includes only monomeric and polymerized actin as well as an inhibitor of actin polymerization (Yochelis et al., 2020). This model was derived from a more detailed model of CDRs in fibroblasts (Bernitt et al., 2017). Its simplicity allows for in-depth mathematical analysis (Yochelis et al., 2022). A more complex variant of this approach has been proposed by Wasnik and Mukhopadhyay, 2014, where different forms of actin monomers and autocatalytic dendritic actin growth are included, as well as negative feedback via coronin that binds to F-actin and promotes its disassembly.

In summary, Figure 3 displays several circuits associated with actin waves in various species including (A) neutrophils, (C) *C elegans*, *Xenopus*, and starfish oocytes, and (E) *D. discoideum*. Notably, common features of these circuits, shown in (F), include the following: (i) the main variable is associated with F-actin assembly or F-actin itself. (ii) The same species creates autocatalytic positive feedback (curved green arrow), enhancing its own activation or assembly from the reservoir. (iii) The main variable promotes some effector that creates negative feedback, generally on a slower timescale. It is well known that such positive and negative feedbacks can give rise to propagating waves, though the details of the patterns and their regimes of existence depend on the specific model. Note, however, that the key motif of these networks is quite general and robustly persists also in the simpler toy models, namely the autoactivation of a key player coupled to slow damping via negative feedback from its product (see also Figure 2A–C).

### Models with dynamic actin network structure

Unlike typical reaction-diffusion models, where the actin is treated as a density variable, models have been proposed that describe the microscopic actin network structure and dynamics in greater detail. Some of these models keep track of polarity fields associated with the actin filament density and orientation (Dreher et al., 2014), or maintain separate densities for the plus (polymerizing) and minus (depolymerizing) ends of the filaments (Doubrovinski and Kruse, 2011). In these models, actin waves are affected by the internal flow of polar actin filaments due to treadmilling along the substrate-attached cell surface. However, the actual wave-generating mechanism turns out to be a combination of cooperative binding of nucleation promoting factors (NPFs) and negative feedback due to inactivation by the local actin filament density, as depicted in Figure 3B. In addition, this model includes the distribution of forces along the cell edge resulting from pushing by actin plus ends, boundary deformation, and dissipative forces. Altogether, this leads to shape changes and net motion of the ‘model cell.’ Note that a similar wave generator based on cooperative nucleator activation and negative feedback from actin (in a classical reaction-diffusion framework with a scalar actin density) was implemented in a simpler cellular Potts model representation of cell shape and motion (Liu et al., 2021).

Active fluid models were also proposed to describe actin wave dynamics. Camley et al., 2013 used a reaction-diffusion-advection system for the concentrations of two components (an actin promoter and myosin, with and without active fluid flow) to display a variety of migration behaviors in a model cell, including periodic directional reversals, coupled to oscillations in the spatial localization of the two components. But even in the absence of reaction-diffusion dynamics, actin waves may be described in terms of an active fluid (Le Goff et al., 2016). This approach does not require any nonlinear biochemistry and solely depends on actin polymerization dynamics, steric repulsion between actin filaments (which tends to align them nematically), and treadmilling. The filaments’ alignment destabilizes the isotropic phase and induces transient actin spots or spirals as part of a dynamic pathway toward a polarized phase, which can be either uniform or may consist of a series of actin-wave trains.

Models that treat the dynamic actin network at the single-filament level may also exhibit wave dynamics on the cell membrane. In one of the earliest models, actin filaments are nucleated by NPFs and are assumed to branch, break, and bind to the membrane (Carlsson, 2010). This model includes a negative feedback from F-actin to the NPFs, as shown in Figure 3B (but without the green positive feedback arrow). Stochastic computations of this model display traveling waves, moving patches, as well as fluctuations, but no moving pulses or periodic structures. Cooperative interactions of NPFs were later added to a continuum version of this model, resulting in more exotic spatiotemporal patterns and waves (Holmes et al., 2012). This model thus includes both positive and negative feedback as shown in Figure 3B, and has later been studied in more detail by LPA (Liu et al., 2021).

Detailed models at the filament level were also proposed for lateral propagation of actin waves along the cell edge (Enculescu et al., 2010; Gholami et al., 2012). Actin polymerizes against the membrane, pushing the cell edge forward. The interplay between polymerization and crosslinking of the filaments into a gel-like network may result in an unstable oscillatory regime that exhibits laterally propagating waves at the cell edge, similar to those observed in experiments (Döbereiner et al., 2006; Machacek and Danuser, 2006; Barnhart et al., 2011; Driscoll et al., 2015). Some models combine several modules, including the chemical network of reaction-diffusion equations, in addition to a filament-scale description of the actin network dynamics (Nonaka et al., 2011).

On the one hand, models that describe the details of the actin network at the level of individual filaments, branching, capping, and cross-linking proteins have the obvious advantage of providing a description that is much closer to the experimental system than the more simplified, reduced models. This includes, for example, the lateral dynamics of the branched network in response to the local topography of the membrane and the effects of bundling (nematic) interactions. On the other hand, tracking details of the actin network components significantly increases the model size and requires greater computational resources. Also, such detailed models are not amenable to mathematical analysis from which insights about the dynamic mechanisms can be obtained.

### Membrane confinement, deformations, and mechano-chemical feedback

The reaction-diffusion approach has also been extended to incorporate the impact of mechanics and membrane deformation. Unlike molecular players, which may be cell-type specific, most of the physical mechanisms reviewed here apply to all cell types.

The confined geometry and the dynamically evolving boundary of a cell may affect the actin wave dynamics, for example, by altering the local density of membrane-bound components of the waves. Deformations of the membrane can also impact the wave patterns by changing the diffusive transport along the convoluted membrane. Furthermore, the local density of cytoplasmic compounds can be affected by membrane deformations, especially when confined, narrow spaces are formed. In a related, but distinct phenomenon, it is well known that the boundary curvature of domain edges can ‘trap’ peaks in a closed (‘no flux’) reaction-diffusion system (Wei, 1999; Iron and Ward, 2000; Bates and Jin, 2014). Examples of this kind in the context of models for a motile cell are shown in Vanderlei et al., 2011; Marée et al., 2012. Furthermore, the dynamics of a reaction-diffusion system can be accelerated when those peaks interact with and change the curvature of a no-flux boundary (Marée et al., 2012; Camley et al., 2017).

Most actin wave models that take the cell geometry into account are two-dimensional projections of the cell, where the cell edge is given by a closed contour. The enclosed area represents the ventral and/or dorsal membranes (or a flattened ‘sandwich’ of membrane and cytosol) on which the actin waves evolve and propagate. Upon collision with the border, a wave may deform the cell contour due to actin-driven protrusive forces (Doubrovinski and Kruse, 2011; Taniguchi et al., 2013; Alonso et al., 2018; Miao et al., 2019; Imoto et al., 2021). Some variants of this approach restrict the reaction-diffusion dynamics to the enclosing contour (Neilson et al., 2011; Shi et al., 2013) or describe the entire deforming membrane in three dimensions (Cao et al., 2019a; Hörning and Shibata, 2019; Winkler et al., 2019; Honda et al., 2021), even taking topological changes due to fusion events into account (Saito and Sawai, 2021).

When cells change shape, cytoplasmic fluid flows are generated by the movement and deformation of the membrane. These flows advect the soluble (cytosolic) components involved in the wave-generating mechanism, affecting their spatial distribution and, consequently, the wave dynamics. Models that incorporate these effects are computationally intensive since they need to resolve the fluid flow field in two-dimensional cell projections or even in three dimensions (e.g., see Campbell and Bagchi, 2018). Alternatively, the cell interior can be described as an active poroelastic two-phase medium, as has been proposed for the protoplasm of *Physarum polycephalum*, where waves of contractile stress propagate through an actin network (Radszuweit et al., 2013; Kulawiak et al., 2019).

A further layer of complexity arises in models that explicitly couple membrane deformation and/or tension to the reaction-diffusion dynamics. Such models often include curvature-sensitive proteins that bind and unbind from the membrane with rates that depend on the local membrane shape. For example, Tamemoto and Noguchi, 2020 and Tamemoto and Noguchi, 2021 showed how curvature-sensitive proteins modify the patterns of propagating waves, even in the absence of actin-induced forces. In another example, Wu et al., 2018 demonstrated how the local membrane deformation modifies the reaction-diffusion dynamics of actin waves via a curvature-sensitive protein that, in turn, affects the Cdc42-N-WASP interaction.

Actin waves can affect and also be affected by membrane curvature. Clearly, actin-driven protrusive forces can locally deform and bend the cell membrane. The resulting curvature can, in turn, affect the dynamics of reaction-diffusion processes distributing actin regulators (e.g., see Marée et al., 2012; Camley et al., 2017). Furthermore, curvature-sensitive membrane proteins that induce actin polymerization (Gov, 2018) contribute to a feedback between curvature and F-actin assembly leading to actin waves. For convex-shaped proteins, which induce an outward bending of the membrane, actin polymerization triggers a local protrusive force that further deforms the membrane, resulting in a positive feedback. The negative feedback that is required for wave propagation can arise in various ways. For example, myosin-driven contractility that retracts the membrane locally may provide a negative feedback by inducing a local concave curvature, causing a lateral shift in the accumulation of the membrane proteins (Shlomovitz and Gov, 2007). Alternatively, negative feedback can also form from a mixture of convex and concave proteins that accumulate in regions where the membrane retracts inward (Peleg et al., 2011). The negative feedback can even arise from external rigid confinement (Naoz and Gov, 2020), which is relevant for actin waves on the ventral cell side, facing the substrate. Finally, a mechano-chemical feedback through Ca^2+^-ion flux can also lead to waves and oscillations (Veksler and Gov, 2009).

Aside from membrane curvature, the feedback via membrane tension has also been considered to couple the actin wave dynamics to the membrane shape. Here, the actin network drives membrane deformations and may thus alter the membrane tension and rigidity, which, in turn, affects the actin network (Wu and Liu, 2021). For example, it was found that membrane tension, which is greatly increased by actin-driven membrane protrusions, can inhibit actin polymerization at the membrane (Houk et al., 2012; Diz-Muñoz et al., 2013). Also the tension-induced release of molecular factors, phospholipase D2 (PLD2) and the mammalian target of rapamycin complex 2 (mTORC2), may inhibit local actin polymerization activity (Diz-Muñoz et al., 2016). This negative feedback couples the membrane deformation to the reaction-diffusion dynamics and, thus, may alter the propagation the actin waves along the membrane. Mechano-chemical feedback that produces actin excitability and wave generation is also at the core of a model for propagating cell-edge actin waves (Barnhart et al., 2017). Here, a membrane-bound actin nucleator (VASP) plays a key role: Local depletion of VASP from the leading edge due to the formation of cellular adhesions, combined with lateral propagation of protrusion due to the branched network architecture, and a global negative mechanical feedback, results in regular waves. But the actin wave itself also applies forces that deform the cell so that, in addition, the reaction-diffusion components of the actin waves may modify the mechanical properties of the cell (Tarama et al., 2022). See also Bailles et al., 2022 for a recent review of mechano-chemical feedback in cells and tissues.

To conclude, models where membrane deformations and tension play a crucial role in the feedback that drives the wave propagation are distinct from the conventional reaction-diffusion models, where actin polymerization/depolymerization is treated as a chemical reaction without mechanical effects. Such mechano-chemical models that we summarized in this section fall into different overall classes. Firstly, some models take the impact of geometry and confinement on the reaction-diffusion-type actin patterns into account either as a downstream effect or by including feedback via curvature sensitive proteins. Secondly, we may distinguish mechano-chemical models, where the mechanical forces that are generated by actin polymerization are an integral part of the wave-generating feedback loops. Here, actin polymerization induces membrane deformations that couple back to the actin activity, for example, via curvature-sensitive membrane proteins or membrane tension. While the mechanical components included in these models are quite universal (due to their mechanical nature), there are also cell-type-specific components (such as curved proteins) that couple them to drive the wave propagation. These models demonstrate that, besides typical reaction-diffusion mechanisms, waves may also rely on nonlocal mechano-chemical feedbacks, and it is not always clear which of these routes to actin waves underlies the experimentally observed phenomena.

Actin waves are observed across different cell types, they are associated with a variety of cellular functions, and multiple wave-generating mechanisms have been proposed, some based on conventional reaction-diffusion dynamics, others involving mechano-chemical coupling. At the current stage of research, it is not clear whether the role of actin is conserved across the wide variety of wave phenomena in different species. More generally speaking, it remains an open question whether a generic theory of actin waves exists or whether theoretical models of wave phenomena in different cell types require principally different mathematical structures. To advance our insight into this fundamental question, further extensions of theoretical methods are required, as will be introduced in what follows.

## What type of theory do we need?

As we demonstrated in the previous sections, many models implement similar ideas with very different levels of detail. While most models of actin waves have been designed to exhibit oscillatory or excitable dynamics, their fidelity to specific experimental observations varies significantly, from phenomenological to realistic aspects, as also indicated in Table 1. Beyond biochemical reaction networks, which are typically described by rate equations, a.k.a. coupled ordinary differential equations (ODEs), transport and mechanical coupling elevate the level of complexity due to space dependence that increases the number of degrees of freedom (Cross and Hohenberg, 1993). In cases where space is of interest, such as for propagating actin waves, the ODE formulation is extended to partial differential equations (PDEs) (Murray, 2001; Baker et al., 2008).

In general, it is impractical, if not impossible, to specify all the processes involved or to obtain a complete set of measurements of rates, concentrations, and interactions within the cell. Alternatively, we seek to discover which of the cell components play critical roles in the observed phenomena. The challenge is finding a compromise between models that have enough ‘realism’ to capture actual biology (e.g., can be related to known cell components) while being ‘simple enough’ to permit in-depth mathematical analysis. This wide spectrum of possible model designs raises fundamental questions to which mathematical theory can contribute: What aspects of the emergent behavior depend on details of a proposed model and what is generic, that is, independent of model details? And is it possible to develop a general theory of intracellular wave dynamics that is still useful to biology? Essentially, any model represents an approximate description of a set of observations, which is often a system’s response upon variation of a controlled quantity. However, the validity of a model is not solely determined by its fidelity (fitting) to the data but also by the ability to predict novel features of the system.

The advantage of simple models with few core building blocks is that they are amenable to mathematical analysis that can reveal the wave generating mechanism. The analysis can then give insights into basic (model-independent) mechanisms of pattern formation even though such models are often far from specific biological interpretations. It is the role of mathematical theory to uncover these core building blocks also in more complex, realistic models that are derived from mechanistic knowledge about the system. Moreover, such core building blocks often represent the more general and robust properties of the system that, in some cases, may be universally valid for a class of similar phenomena, such as actin waves in different biological species.

Previously, we reviewed different modeling approaches. In what follows, our aim is to introduce methods from the so-called bifurcation theory of nonlinear PDEs that provide a framework to identify the core building blocks of complex biological models. Through examples from the field of actin waves, we also demonstrate the methodology’s strength. We emphasize that the purpose is to provide a descriptive picture rather than a rigorous mathematical review, while organizing the more technical details, for math-inclined readers, in focused boxes.

### Mathematical methodology for actin waves

In the 18th century, Laplace was convinced that Newtonian mechanics is a sufficient methodology to uncover the ‘secrets’ of the universe, “Give me the positions and velocities of all the particles in the universe, and I will predict the future.” It took, however, another century until Poincaré demonstrated the inherent limitation of Newtonian (linear) mechanics arising already in the (nonlinear) motion of three interacting particles. In contrast to Laplace’s philosophy, Poincaré showed that such motion cannot be predicted (Poincaré, 1885), laying the foundations of what was to become the so-called chaotic dynamics as well as bifurcation theory, in general.

The essential difference between linear and nonlinear systems is the number of possible solutions: While linear models have only one solution, nonlinear models may admit coexisting solutions, which can be uniform or may vary in space and time. Furthermore, the stability properties of solutions are important as they affect sensitivity to initial conditions and to perturbations as the system evolves in time. However, uncovering the evolution of multivariable nonlinear PDEs is a paramount and ongoing mathematical challenge. Thus, in many cases, we rely on in silico simulations to describe complex systems (e.g., biological, chemical, and ecological). These simulations are numerical experiments rather than theoretical frameworks. In what follows, we briefly survey and exemplify the *bifurcation methodology* that is, to date, the most powerful approach to analyze the solutions of nonlinear PDE systems.

#### Fundamentals of bifurcation theory at a glance

Bifurcation theory has been developed to analyze dynamical systems described by ODEs (Strogatz, 2018). The term ‘bifurcation’ stems from Latin and describes a branching or splitting. In the context of dynamical systems, it was introduced by Henri Poincaré and refers to the branching of solutions (Poincaré, 1885). Mathematically, a bifurcation point corresponds to a sudden qualitative change in the system’s behavior that is related to a transition between different states of the system or to an exchange of a state’s stability, as a control parameter is varied.

**Figure 4.**
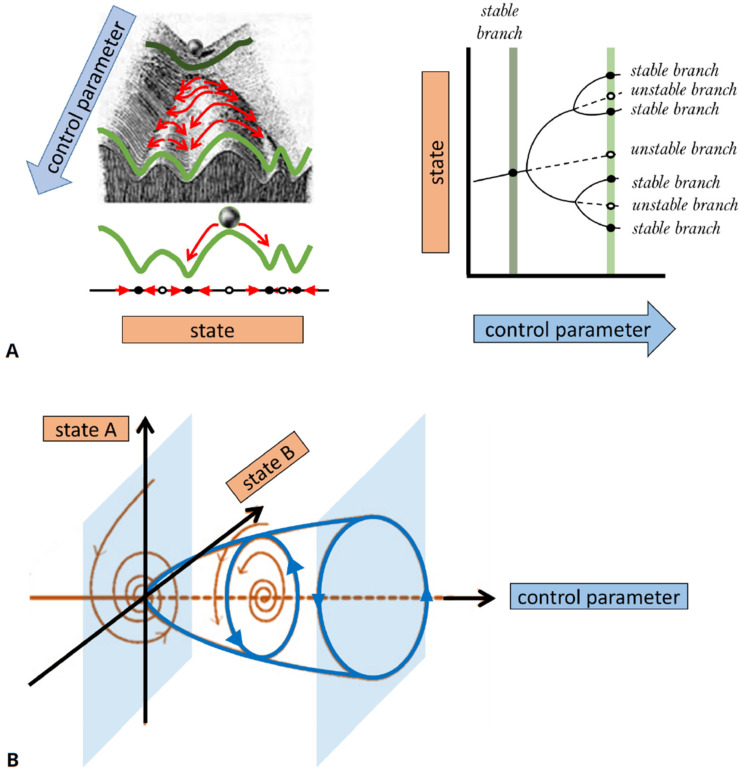
A basic description of bifurcation methodology. (**A**) Phenomenological description of a bifurcation diagram using the ‘Waddington landscape.’. Left panel: The ‘state’ of a system is described by a single variable (axis labeled ‘state’). The evolution of the system with time (red arrows) is away from unstable states (open circles and mountain tops) and toward stable states (black circles and valleys). The directions of changes over time can be compressed into a flow diagram. The effect of a control parameter is shown as a shift in the geography of the hills and valleys. Right panel: A bifurcation diagram containing the information corresponding to the ‘Waddington landscape,’ where a stable state (solid line) becomes unstable (dashed line) to a pair of new stable states, a so-called ‘pitchfork’ bifurcation. The solid (dashed) lines correspond to valleys (mountain tops) in the left panel. The ‘bifurcation points’ mark parameter values, where small changes in a control parameter result in qualitative changes in the system’s behavior, e.g., when new solutions emerge at a branching point or their stability is changed. Bifurcation analysis allows us to map the ‘landscape’ of the underlying dynamical system and elucidates how various regimes of different behavior are related. In general, some branches of the bifurcation diagram may represent persistent oscillations, waves, or pulses in the system’s behavior. In (**B**), we schematically show a bifurcation to periodic oscillations (a.k.a. limit cycle solutions) for which the amplitude increases with distance from the bifurcation onset. Such instability is also known as a Hopf bifurcation and requires a system of at least two state variables (as opposed to a steady state pitchfork bifurcation demonstrated in (**A**) that may arise in models with a single variable). The shaded surfaces depict two selective phase-space projections spanned by the state variables at specific values of the control parameter, where the arrows correspond to temporal dynamics.

Conventionally, bifurcations of solution branches are described in diagrams that show the effect of changes in control parameters on the existence of solutions and their stability. (For example, a one-parameter bifurcation diagram resembles a tree-like structure, as shown in the right panel of Figure 4A.) Stable (unstable) solutions are typically depicted by solid (dashed or dotted) curves. A bifurcation diagram can be thought of as a ‘landscape’ on which the system evolves, similar to a ‘potential surface’ in physics or the ‘Waddington landscape’ in developmental biology. In Figure 4, we show a schematic example of many possible behaviors by a mathematical model, the dependence of that behavior on a control parameter, and the values of the parameter at which the behavior changes. The main ‘branches’ of this diagram represent the system’s behavior after transients are gone, and the system has settled into one of its stable regimes. We elaborate further on the concepts of stability and classifications in Box 3.

The appearance and/or stability of these solutions obey specific mathematical rules, for example, past a bifurcation point, an increase in the number of solutions is always by pairs (one stable and one unstable), while if the number of solutions remains the same, then their stability is exchanged (i.e., the stable becomes unstable and vice versa). Different bifurcation scenarios can be distinguished. They represent model-independent behaviors near the bifurcation onset. In Figure 4, we describe two representative cases of a ‘pitchfork’ and a ‘Hopf’ bifurcation. A pitchfork bifurcation represents the formation of two coexisting stable states (a regime of bistability) out of one stable state, as a function of a control parameter. A bistabile regime is observed, for example, when reactants undergo a chemical reaction that results in a mixture of two chiral molecules (enantiomers). Both enantiomers are stable and may coexist, while the initial non-chiral reactants are unstable. Also in many biological systems, multistability is observed, for example, when cells differentiate into different coexisting types of tissue or ecosystems converge to different compositions of stable coexisting populations or vegetation types. A Hopf bifurcation describes an onset of persistent time-periodic oscillations (often referred to as ‘limit cycle’ solutions). They are ubiquitously observed in biological systems, for example, in the regular pacing activity of the sinoatrial node in the heart, the circadian rhythms, the Rho activity in early *C. elegans* embryos, the genetic clock in somitogenesis, or cyclic adenosine monophosphate (cAMP) dynamics in developing *D. discoideum* cells. Such cyclic dynamics require at least one activating species with autocatalytic or enzymatic steps and an inhibiting species that evolves at a slower timescale.

Basic bifurcations, such as the pitchfork and Hopf bifurcations in Figure 4, are governed by variation of a single control parameter and are termed co-dimension one bifurcations, while more complex bifurcation scenarios may require the tuning of several control parameters and are, thus, termed as higher co-dimension bifurcations. In general, the higher the co-dimension of a bifurcation, the richer the qualitative accompanied dynamics as well as the sensitivity to initial conditions. Consequently, studying high co-dimension bifurcations may reveal the core building blocks of a model and allow the design of specific tests to differentiate between different dynamic behaviors in experiments. For further details, we refer the reader to introductory (Strogatz, 2018) and to more advanced texts (Crawford, 1991; Kuznetsov, 2004; Guckenheimer and Holmes, 2013).

Box 3. **Linear vs. nonlinear bifurcations**

In this box, we briefly overview the concept of stability. For illustrative purposes, consider a cell that could be rounded (state A) or spread out (state B). A state is said to be linearly stable (or unstable) if a small superimposed disturbance or ‘perturbation’ decays (or grows) with time. We consider both static (steady state) and periodic (limit cycle) cell behaviors (e.g., a cell that oscillates between contracted and spread). Mathematically, we can calculate quantities that diagnose stability. These values are called eigenvalues (for steady states) and Floquet multipliers (for limit cycles).

As a control parameter is tuned past some ‘bifurcation value,’ the stability of a system may change. We distinguish between super- and subcritical bifurcations. In the former, arbitrarily small disturbances (like natural thermal fluctuations) would destabilize the system. In the latter case (i.e., when moving along the linearly stable solution toward the bifurcation point), only a sufficiently large disturbance (larger than some predictable threshold) would do so. For example, a small stimulus might fail to provoke a cell to round up or spread while a sufficiently large stimulus would do so, as shown in Box 3—figure 1A. Coexisting states (as in the case of A and B in Box 3—figure 1B) also generically come with a property called hysteresis, if two or more bifurcation branches are connected.

We exemplify this with the bifurcation diagram displayed in Box 3—figure 1B. In the region of stably coexisting states A and B (shaded blue), only a threshold stimulus causes state transition. Outside of this parameter regime (white), only state A or B exists. Tuning the control parameter up (horizontal green arrows) will eventually induce a transition from state B to state A (vertical red arrow). But it then takes considerable ‘detuning’ of the same parameter to get back to state B (red horizontal and green vertical arrows). Thus, the state transition depends on both state and history. We refer to the full excursion as a hysteresis loop. This is the mechanism that leads to coexisting oscillations and pulses when an excitable element is coupled to slow negative feedback from its ‘refractory’ partner variable (Kobayashi et al., 1994; Kobayashi et al., 1995). It can also explain periodic cell rounding-spreading if myosin activity (causing contraction) is coupled to a mechanosensitive cell element (sensing contraction) that suppresses myosin activity (e.g., see Zmurchok et al., 2018).

**Box 3—figure 1.**
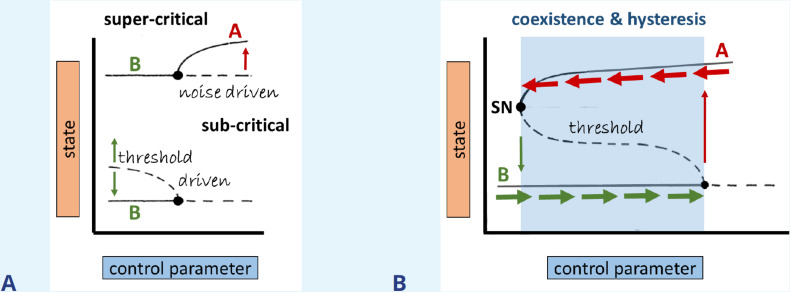
Bifurcation types, coexistence, and hysteresis. (**A**) Illustration of super- vs subcritical bifurcations, where the thin red/green arrows mark the direction of the dynamics with respect to time, respectively. (**B**) Coexistence (bistability) is formed via the connection of branches emerging from the subcritical and saddle-node (SN) bifurcations, as indicated by the shaded region. Thick horizontal arrows indicate at which state the system persists for perturbations that do not exceed the threshold (dashed line).

The direction of the bifurcating solution branches, as shown in Box 3—figure 1A, is important. This distinction, termed super- and subcritical bifurcations, allows insights into how the evolution of the system depends on its initial conditions or on perturbations. In the super-critical case, a stable solution loses stability beyond the bifurcation point (‘at onset’ of the bifurcation), where even small noise can trigger a spontaneous transition to a different state of the system. The subcritical case is identified with threshold behavior since, before onset, the bifurcating solutions are unstable, as shown in Box 3—figure 1A to the left of the black dot. We refer to this as a nonlinear bifurcation. To switch to a new solution, perturbations have to be large enough (above threshold) to drive the system across the unstable branch (dashed curve shown in Box 3—figure 1). The subcritical case is thus associated with finite (as opposed to infinitesimal) perturbations. Also, it is often accompanied by hysteresis, where the system’s behavior is history-dependent, switching between states at different critical parameter values, depending on the direction in which the control parameter is varied (Box 3—figure 1). An intriguing biological example of experimentally observed hysteresis occurs in Rac-Rho-PAK signaling in breast-cancer cells as described in Byrne et al., 2016. In physics, hysteresis is associated with first-order phase transitions, for example, in super-cooling, where liquid water persists even below the freezing temperature.

While bifurcation diagrams are a highly informative summary, they can be challenging to construct and validate. Experimentally, one would have to run a controlled experiment many times, varying a single parameter (e.g., titrating the concentration of an inhibitor in small steps, both up and down in values) to observe whether transitions in behavior take place and where. (Recall that within a hysteresis region, gradual increase or decrease of a parameter results in transitions at *different* parameter values.) For a two-parameter bifurcation plot, even more experiments would be required, varying each of the two key parameters. This is prohibitively expensive in time and material, meaning that few such diagrams are found in purely experimental papers. Relying on a theoretical model, we can generate bifurcation diagrams ‘more easily’ since the dynamics can be simulated over and over again to produce even two-parameter bifurcation diagrams, as done, for example, in Arai et al., 2010; Shibata et al., 2012; Bolado-Carrancio et al., 2020; Staddon et al., 2022. With fast computers, this is a possible avenue that requires no sophisticated mathematical tools, yet, it is also challenging for several reasons:

- For realistic models, there are typically many control parameters, for example, N>10 in Michaud et al., 2022. Identifying a subset of two parameters to explore, and values for each of the remaining N-2, is a nontrivial task. Evidently, such parameter spaces are huge, and even well-informed searches in parameter space can fail to find the regimes of interest. Hence, brute-force computations can be frustrating and ineffective.
- Even after successfully arriving at the desired numerical bifurcation plot, the insights gained by such methods are limited, for example, it may be still difficult to determine the robustness of the model structure (i.e., the persistence of qualitative features in the dynamics if reaction terms are changed or upon addition of another variable). The benefit of leaning on rigorous methods instead is that they not only help to find the interesting transitions and relevant regions in parameter space, but also ‘peal away the layers of complexity’ close to those transitions.

Importantly, bifurcations may also act as *organizing centers*, affecting nearby dynamics, meaning that in the vicinity of such bifurcations, there exist additional behaviors that are also model-independent, and that are recognizable as generic ‘signatures’ of more complicated dynamics. This is particularly relevant in the case of so-called *global* bifurcations since the dynamic flow about these bifurcations is not limited to the proximity of a specific solution branch. In contrast to local bifurcations, however, analysis of global bifurcations requires deeper mathematical knowledge.

**Figure 5.**
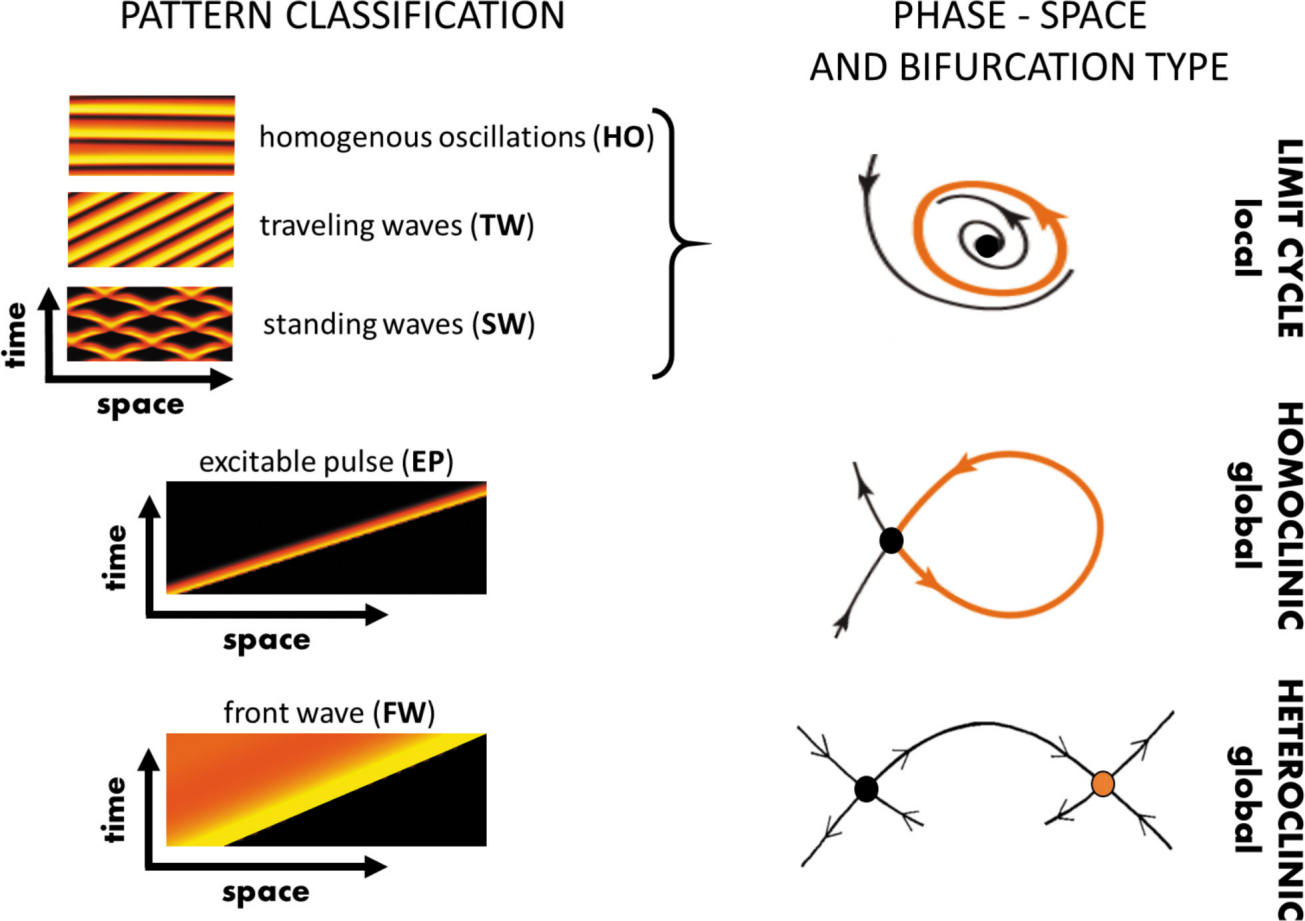
Various realizations of propagating waves and pulses. Left panel: Three classes of propagating (time-dependent) patterns: Oscillatory waves, an excitable pulse, and a front wave (a*.*k*.*a*.* a traveling front); orange and black colors correspond to maximal and minimal values of the solutions, respectively. Right panel: The spatial variation of key variables across a wave (e.g., the variation in fluorescence intensity from front to back) can be represented by a trajectory in some high-dimensional ‘phase space’ spanned by the model variables (here simplified to a 2D cartoon) connecting the back state to the front state (see text), or varying periodically across the wave. Dots correspond to steady states and arrows describe flows in their vicinity. Classification of the associated local/global bifurcations through which such solutions form. Note that in phase space various oscillatory solutions all have the same geometric flow, described by a limit cycle (periodic orbit).

As an example, let us consider the characterization of patterns such as broad moving fronts versus localized (static or moving) pulses (see Figure 5). For example, an actin nucleation promoting factor (NPF) moving as a broad front could lead to wide, F-actin-rich protrusions, whereas in the latter case, pulses of NPF may engender localized F-actin sites such as neuronal dendritic spines. Such distinctions have clear significance to cell behavior. Now consider the key differences in these patterns: The front is a wave with values of NPF that vary over space from a low- to a high-density state across the wave (front to back), while the pulse corresponds to a variation from low to high and back to low NPF density. Mathematically, these waves are often depicted in so-called ‘phase-space’ diagrams as trajectories connecting states (see Figure 5). For example, *heteroclinic* bifurcations connect coexisting states while a *homoclinic* bifurcation is a closed loop connecting a state to itself. We can use mathematics to characterize the appearance or disappearance of these waves (and their speed) as global bifurcations, where trajectories of one kind (e.g., homoclinic, for a pulse) get broken and replaced by others (e.g., heteroclitic, for a front). As such, solutions emerging near global bifurcations are associated with dynamic trajectories (manifolds) that are generally far from any bifurcation onset and thus, their qualitative properties are generic (model-independent or *structurally stable* in a mathematical sense; Guckenheimer and Holmes, 2013). A prominent example is a propagating excitable pulse depicting an action potential in neural and cardiac systems (Murray, 2001; Murray, 2002; Keener and Sneyd, 2009). These solutions display a finite amplitude excursion, starting from a rest state and decaying back to the same state (i.e., a homoclinic loop). In contrast, the heteroclinic trajectories represent fronts that are associated, for example, with the invasion of one state into another, where the states may represent different species, concentrations, or cell types.

Next, we elaborate on the key role of global bifurcations in identifying robust features of wave propagation phenomena, relevant to our understanding of actin waves.

#### Bifurcations and waves in spatially extended systems

After describing the basic ideas underlying the bifurcation theory of ODE systems, we now take into account space dependence that may arise due to transport and mechanical coupling. Mathematically, this is reflected in PDE-type model equations that inherently have infinite degrees of freedom due to spatial modes and dependence on boundary conditions. There are several rigorous methodologies for PDE analysis (e.g., multiple timescale reduction and singular perturbation theory). However, similarly to the phase-space method, also other advanced reduction methods can be efficiently applied in a few simple cases of two-variable toy models expressed via polynomial-type terms that mimic the realistic interactions, such as in the FitzHugh–Nagumo, Schnakenberg, and Gray–Scott reaction-diffusion systems. More involved and detailed models of actin dynamics are typically studied by direct numerical integration. Consequently, lacking theory, it is difficult to determine which of the solutions are sensitive to model details and which are robust (structurally stable).

Even though bifurcation theory for spatially extended systems is significantly less advanced than for ODEs (Cross and Hohenberg, 1993; Sandstede and Scheel, 2000; Pismen, 2006; Meron, 2015), in some cases, mathematics can still provide valuable insights. Such cases often rely on specific properties of solutions such as traveling waves or pulses, where PDEs can be reduced to ODEs that describe the (fixed) shape of a solution along a transformed coordinate (say ξ), instead of the spatiotemporal dependence (e.g., using a comoving frame transformation such as ξ=x±v⁢t, where v is the wave speed). One then seeks bifurcations that depict the onset and demise of such wave patterns. Following these ideas, most of the propagating actin patterns can be classified (using phase-space reduction) according to their oscillatory, excitable, or bistable properties (cf. Allard and Mogilner, 2013). Figure 5 summarizes these three classes; note that we exclude here the transient actin polymerization dynamics that converge to steady-state patterns, as discussed in Bhattacharya et al., 2020.

However, these classifications do not resolve the origin and the mechanisms of the emerging actin waves. For example, many of the observed actin patterns are of a spiral form. Yet, since spirals share universal properties (Sandstede and Scheel, 2020) and can form in oscillatory, excitable, and bistable systems, it is difficult to decipher to which dynamic class the system belongs. Moreover, there are also basic subtleties that are frequently overlooked when relying on ODE classifications, such as secondary (space-dependent) instabilities (Cross and Hohenberg, 1993). This directly impacts attempts to determine the lowest number of variables needed to describe a certain phenomenon in a qualitative (generic) manner. For example, as has been shown in Figure 4B, in ODEs a steady state can lose stability to oscillations via the so-called Hopf bifurcation while in PDEs, oscillations can arise via two different mechanisms, leading to three qualitatively different patterns. The first type is the natural extension to homogeneous oscillations (HO), a.k.a. Hopf bifurcation with a zero wavenumber (infinite wavelength since a wavenumber is proportional to the inverse of a wavelength). As for ODEs, also for PDEs the minimal setting is a two-variable system. The second type is a Hopf bifurcation with a finite wavenumber (or finite wavelength), where the resulting oscillations are counter-propagating traveling waves (TW) and standing waves (SW); for more details, see Knobloch, 1986. In Figure 5, we display these three oscillatory patterns (HO, TW, SW) in a spatially extended system. In contrast to the zero wavenumber Hopf bifurcation, the finite wavenumber Hopf bifurcation requires a three-variable system (Yochelis et al., 2008; Stich et al., 2009; Anma et al., 2012; Hata et al., 2014; Villar-Sepúlveda and Champneys, 2023). In Box 4, we provide additional heuristic details about the richness of the primary finite wavenumber Hopf bifurcation. More advanced related topics, mixed-mode solutions, secondary instabilities, and conserved quantities are detailed in Knobloch, 1986; Knobloch, 1992.

To summarize, while the bifurcation theory of PDEs is still a developing field, especially in nonlinear regimes, it has matured to study some of the actin wave phenomena, as will be briefly demonstrated by examples in the following. Yet, further extensions are needed, especially in the broad context of mechano-chemical feedback (e.g., see Ben Isaac et al., 2013), where nonlocal interactions, advective flows, and changing geometries are coupled to reaction-diffusion dynamics.

Box 4. **Bifurcations of spatially extended oscillatory solutions**

In Box 3, we showed how oscillatory behavior (limit cycles) can arise in time-dependent systems (ODE models); see also Figure 4B. Adding spatial coupling (PDE models) affects sensitivity to perturbations (e.g., to input stimuli), especially in systems with many interacting components (many model variables) characteristic of most biological systems. Such models can potentially exhibit a vast range of qualitative behaviors that are challenging to characterize and study.

Bifurcations in PDEs are currently only partly understood. Still, we know that oscillations can arise through bifurcations that produce one of several outcomes: Spatially uniform cycles or waves. The latter include traveling (TW) or standing (SW) waves (Figure 5). The birth of spatially uniform cycles is predicted by the same type of (Hopf) bifurcation in PDEs as in ODEs, but the appearance of TW/SW waves is simultaneous and has a bifurcation structure that has no analog in ODEs (a so-called finite-wavenumber Hopf bifurcation). Mathematically, we can predict that TW and SW can appear (emerge at a bifurcation point) in one of six possible ways (both super- and subcritical, see Box 4—figure 1), contrary to only two types of bifurcations for spatially uniform cycles (Knobloch, 1986). As shown in Box 3, subcritical bifurcations imply coexistence of unstable branches over a large range of parameter values. Such waves have a richer sensitivity not only to size but also to spatial forms of stimuli. Moreover, in many mass-conserved systems (e.g. conserved amount of total actin over the timescale of actin-wave formation; Beta et al., 2020), new behaviors can arise (e.g., TW that stall to form a polarized distribution) (Knobloch, 1992). Furthermore, domain size, shape, curvature, and boundary conditions are significant (Yochelis et al., 2022).

Current mathematical methods are not yet sufficiently developed to decipher bifurcation structures that explain the appearance of spatially oscillatory dynamics just from experimental cell images or movies. This is especially true for 2D and 3D deforming cells (motivating 1D ‘cell in a channel’ experiments that simplify the geometry, e.g., Hadjitheodorou et al., 2021). So far, we can use mathematics to exclude some mechanisms. For example, we know that a finite wavenumber Hopf bifurcation to TW and SW cannot arise in two variable RD models (Villar-Sepúlveda and Champneys, 2023). A third component is required to account for trains of pulses emanating from a single localized stimulus (e.g., see Yochelis et al., 2008; Yochelis et al., 2015). (Localized optogenetic activation of NPF results in a sequence of filopodia moving bidirectionally away from the stimulated site.) More exotic dynamics include jumping spatially localized oscillations (oscillons) (Yang et al., 2006; Knobloch et al., 2021), observed, for example, in the pattern-forming Belousov–Zhabotinsky chemical reaction (Cherkashin et al., 2008). Hence, oversimplifying a system may result in loss of fundamental dynamic behavior.

**Box 4—figure 1.**
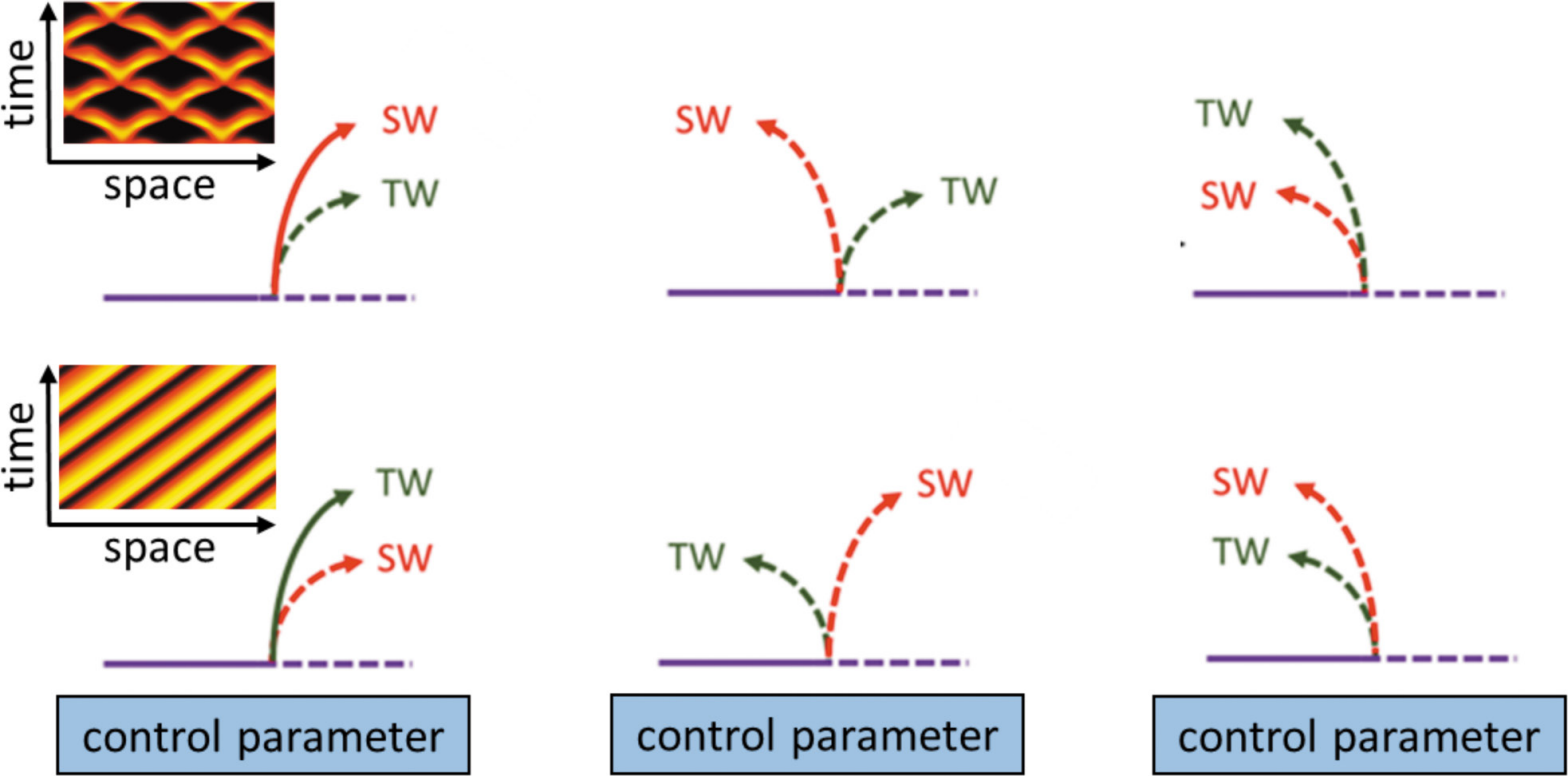
Schematic description of possible ways in which primary bifurcating traveling waves (TW) and standing waves (SW) can emerge from the finite wavenumber Hopf bifurcation**.** Solid (dashed) lines indicate stable (unstable) solutions of a certain state variable (modified from Knobloch, 1986). The left insets demonstrate space-time plots of typical forms of SW employing no-flux boundary conditions (top) and TW employing periodic boundary conditions (bottom) that emerge from super-critical bifurcations.

### Insights obtained using bifurcation theory

#### Linking cell responses to model mechanism

In recent years, new tools have been developed for prodding, stimulating, and manipulating cell behavior, such as optogenetics, where light signals activate one or another regulator in a highly localized manner inside a cell (see O’Neill and Gautam, 2014; O’Neill et al., 2016; Meshik et al., 2018; Hadjitheodorou et al., 2021). This has resulted in rich data sets connecting the strength, size, and timing of the stimulus to resultant cell responses.

**Figure 6.**
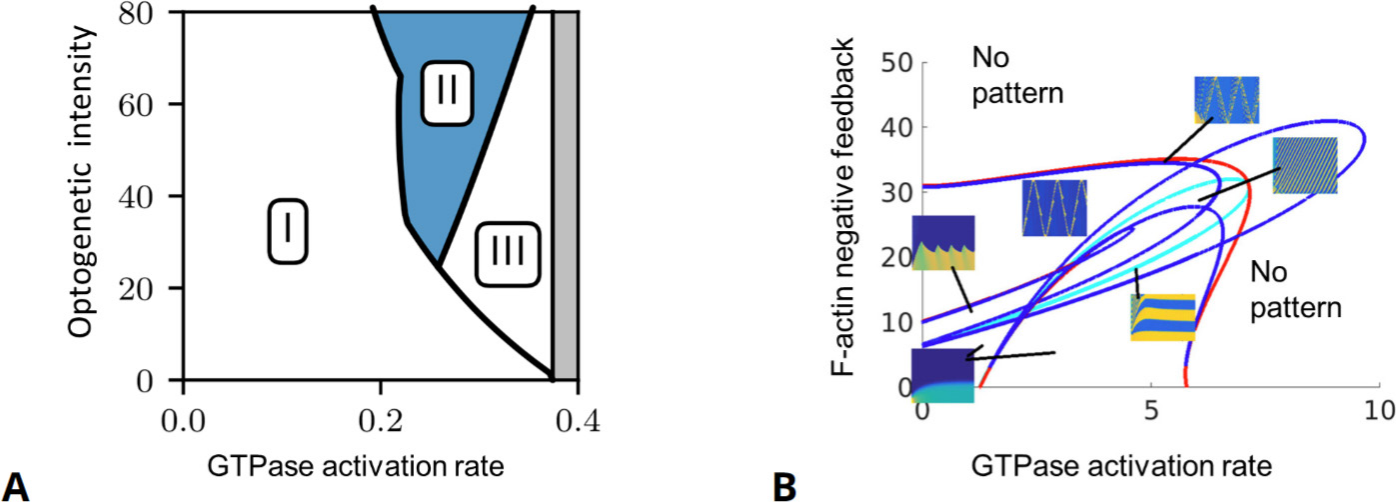
Examples of full and reduced bifurcation analyses. (**A**) Full partial differential equation (PDE) bifurcation analysis of a GTPase model with no F-actin feedback. Shown are regimes of the response of a polarized model cell to a ‘polarity reversal’ stimulus. Horizontal axis: The inherent spatially uniform GTPase activation rate. Vertical axis: Optogenetic stimulus intensity that amplifies that rate in the back of the cell (% increase in GTPase activation rate for a stimulus of width 0.75 cell diameter, see experiments by O’Neill et al., 2016). The regimes represent failure to repolarize (i), reversal of polarization (ii), and loss of polarization (iii). (**B**) A shortcut bifurcation analysis of the actin wave model of Holmes et al., 2012 using local perturbation analysis (LPA). This two-parameter diagram displays borders of the patterning regimes (solid curves), together with typical behavior (kymographs of the full PDE solutions in one-space dimension) inside those regimes. Curves indicate Hopf (blue), fold (red) and transcritical (light blue) bifurcations in the LPA version of the model equations, as described in Liu et al., 2021. Insets: Kymographs showing spatiotemporal nucleation promoting factor (NPF) activity (yellow=high, blue=low) with time on horizontal and space on vertical axes. (**A**) is modified from Figure 6 in Buttenschön and Edelstein-Keshet, 2022 and (**B**) modified from Figure 8 in Liu et al., 2021.

The question is how to understand cell responses from such data sets, and what such responses tell us about the regulatory circuits that govern those responses. For example, in O’Neill et al., 2016, an optogenetic signal was used to repolarize macrophages by photoactivating a Rho-GEF (activating the GTPase Rho at the front of the cell). In some cases, this stimulus will repolarize a cell, whose initial Rho activity was concentrated in the rear. This reversal of polarity was quantified in two models using PDE bifurcation analysis (Buttenschön and Edelstein-Keshet, 2022), mapping out how the signal intensity, coupled to the inherent cell parameters, elicits a response (Figure 6A). In some regimes, the cell does not repolarize (i), in others it loses polarization entirely (iii), while in a wedge-shaped region (ii), it repolarizes in a direction opposite to its initial polarity. The analysis revealed distinct response ‘signatures’ depending on the assumed underlying polarization model. Such synergy between models, analysis, and experimental data can be used to distinguish competing models for the underlying regulatory circuits. The mathematical analysis demonstrated that cells with mutually antagonistic (Rac-Rho) polarity GTPases (as in Holmes and Edelstein-Keshet, 2016) have much simpler routes to repolarization than cells with a single dominant polarity GTPase (as in the wave-pinning model by Mori et al., 2008). It also gave important insights into how cell repolarization takes place. Similar coordination between experiments that provoke dynamic actin waves and mathematical PDE bifurcation analysis of the proposed models could, in future, help to refine our understanding of those dynamic phenomena.

To date, a full PDE bifurcation analysis has not yet been applied to most biochemically regulated actin wave models. The shortcut of LPA has been used to explore how parameters such as basal GTPase activation rate (GEF activity) and F-actin negative feedback (GAP activity) affect the formation of various dynamic patterns (Holmes et al., 2012; Mata et al., 2013). One example of a two-parameter LPA bifurcation diagram from Liu et al., 2021 is shown in Figure 6B. In principle, these predictions can be tested against data that inhibits/overexpresses an F-actin-associated GAP (like RGA3/4) or similarly manipulates the corresponding GEF.

#### Identification of circular dorsal ruffles as front phenomena

As has been shown throughout this review, actin waves are abundant in cells and arise in a wide variety of forms. However, since in experiments these waves are fully developed, that is, the instability at the onset of wave formation is typically not observed, there is an inherent difficulty to classify them and determine whether they arise via the same generic mechanism or whether there are fundamentally different coexisting classes of wave patterns that are generated via distinct biochemical circuits (cf. Michaud et al., 2022).

**Figure 7.**
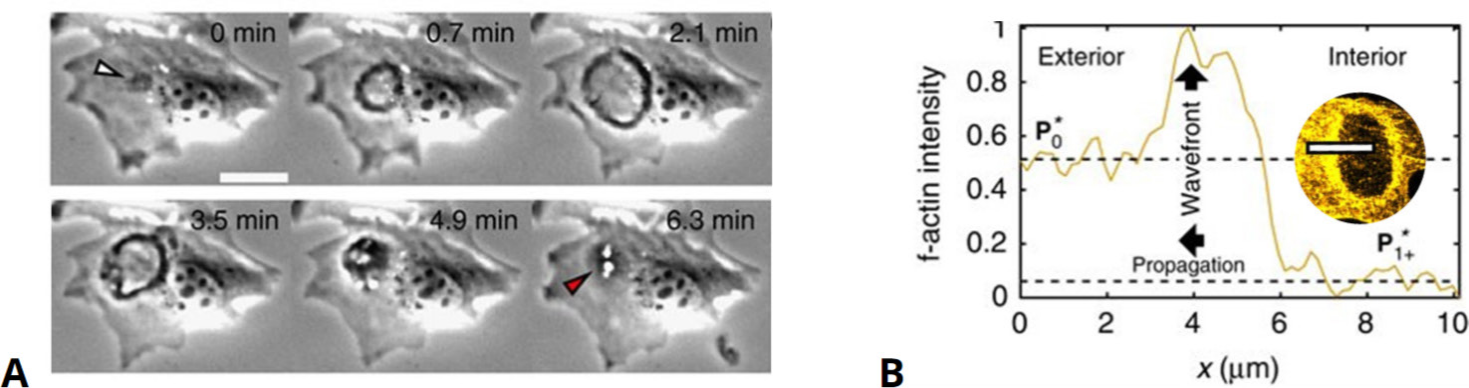
Front dynamics in circular dorsal ruffles. (**A**) Experimental phase-contrast recordings of circular dorsal ruffles (CDRs) showing one cycle of the (dark colored) ring-shaped expansion and contraction. (**B**) F-actin concentration along the propagating wave front upon CDR expansion outward as also indicated by the top view in the inset, respectively. Modified from Bernitt et al., 2017.

CDRs are ring-shaped actin waves on the dorsal side of cells that are related to endocytosis (engulfment of external material). Typical phase-contrast microscopy images of CDRs are shown in Figure 7. As failures in CDR development are believed to be associated with cancerous phenotypes, a mechanistic understanding of their dynamics is of prime importance (Bloomfield and Kay, 2016). Previously, it was assumed that CDRs belong to the class of excitable waves, as annihilation upon collision frequently occurs (Zeng et al., 2011; Bernitt et al., 2015). However, this hypothesis was challenged by the observation of reflections of CDRs at the cell boundary, which are not expected in an excitable system.

This inconsistency has stimulated new research, combining live-cell imaging, mathematical modeling, and bifurcation analysis (Bernitt et al., 2017). Using confocal fluorescence microscopy recordings to quantify the actin density along the dorsal cell cortex, a significant difference in the actin density between the interior and the exterior of CDRs was observed, as shown in Figure 7B. The two regions of different actin density clearly indicate a bistable situations, where both the low and the high actin density regimes are stable solutions of the actin system. Moreover, the actin profile across the CDR border connects actin-rich and -poor regions and, therefore, corresponds to a generic ‘front’ connecting these two states (with an additional localized overshoot at the border). This evidence for bistability motivated a simple mass-conserved reaction-diffusion model in which actin waves naturally arise from the interplay of F-actin, G-actin, cortical actin, and an F-actin inhibitor. An analysis of this model confirmed the reflections of CDRs at boundaries and also accounted for the different propagation speeds of expanding and contracting rings observed in the experiments. They are related to two distinct coexisting front states, a property that naturally arises in bistable systems but is not expected in excitable media. Moreover, bifurcation analysis of front stability that was followed by experiments suggested conditions under which CDR failure can occur, giving rise to either disordered wave dynamics or front pinning at the cell boundary, thus, providing fresh insights into CDR dynamics and regulation. Beyond the mechanistic understanding of CDRs, these results may, in future, allow us to explain the link between the dynamics of CDRs and cancer progression by providing a framework to understand the roles of different cytoskeletal components and signaling factors (Orth and McNiven, 2006; Hoon et al., 2012; Itoh and Hasegawa, 2013).

## Discussion and outlook

Actin waves appear in a wide variety of cell types and conditions. In some but not all such systems, these waves are correlated with certain cell functions, for example, edge protrusion (in motile cells) or cytokinesis. In other systems, the correlation with cell function is less clear, but we can use the existence, shapes, frequencies, and other wave characteristics to understand underlying processes at a more basic level of organization (i.e., the chemical and mechanical interactions that lead to those waves).

As surveyed here, different modeling approaches have been proposed to describe actin waves, some tied directly to experimental findings, and some primarily phenomenological. A challenge has been to strike the right balance between the level of biological detail and mathematical tractability. It is evident that the key driving molecular components may differ from one cell type to another (or between experimental conditions) since some cells or cell states lack effectors that are central in others. However, we often see related dynamic circuits (even in distinct molecular signaling networks), as, for example, in the case of a bistable switch driven around a cycle by negative feedback.

We believe that mathematical methods (including bifurcation analysis) are useful to truly characterize and synthesize the common features and differences between classes of models and, hence, between the distinct mechanisms leading to actin waves. This task, however, is nontrivial given the fact that much of the current model complexity is beyond the reach of existing mathematical methods, emphasizing the need (i) to further develop those mathematical tools and (ii) foster close consultation between biologists and mathematicians to identify the backbone components.

Theoretical investigations of actin waves are still driven primarily by simulations. However, even simulations that demonstrate biologically realistic behavior do not necessarily point at the correct underlying mechanism and mostly fall short of mapping out full regimes of behavior. To substantially advance the field, a grand synthesis of existing models is required, describing the common aspects, and identifying their unique vs. shared features. This type of synthesis would contribute to our understanding of universal (versus specific) cellular mechanisms, conserved mechanisms, and those that arose via convergent evolution in different cell types. Such collaborative work of biologists and mathematicians would also identify a minimal set of models, biologically relevant, and yet sufficiently compact to be analyzed mathematically in full. Needless to say, the analysis and the formulation of novel mathematical methods would also contribute broadly to the theory of PDEs, the physics of active matter, nonlinear chemical reactions/kinetics, and the biology of eukaryotic cells.

We conclude by listing some challenges for future research in this field. First, for the modeling community, we advocate for the development of a comprehensive ‘atlas’ of (small) regulatory modules that can (or cannot) account for observed waves. Here, we refer to generic circuits, whose dynamics have been well-documented, with modifications that remove or add specific behavior. Examples of such work include Kholodenko, 2006, where a combinatorial set of variants of feedback connectivity is mapped out to determine which ones result in a relaxation oscillator. The multiple (small) circuits discussed by Tyson et al., 2003 contribute to a similar flavor, helping to identify specific regulatory circuit topologies with known behavior. We also believe that new models that are proposed should be accompanied by comparisons to those that already exist.

Among the mathematical challenges, we can identify several areas that, in our opinion, merit highlighting:

- Apparently, PDE bifurcation analysis is still a specialty that is less familiar in biological sciences. This merits further development of pedagogical tools to emerge as a ready-to-use method (in contrast to ODE bifurcation analysis, for which many tools for nonexperts exist; e.g., see Strogatz, 2018).
- In communities that work at the border of computational biology, bifurcation theory is frequently employed, however, mostly at the linear analysis level. Yet, since most of the observed phenomena are far from the linear limit, nonlinear bifurcation theory relying on global bifurcations should be advanced to provide interpretations and mechanistic understandings.
- In classical models of biological wave patterns, for example, in the FitzHugh–Nagumo, Gray–Scott, Gierer–Meinhardt, or Schnakenberg models, there are no ‘conserved quantities.’ However, as demonstrated in this review, in intracellular actin waves, some quantities are conserved on the timescale of wave formation (switch-like signaling proteins, actin in various forms, etc.) The effect of mass conservation has been discussed, for example, in Jilkine and Edelstein-Keshet, 2011; Halatek and Frey, 2018; Brauns et al., 2020; Champneys et al., 2021, but its role in wave formation, nonlinear oscillatory instabilities, and pattern selection is still limited and should be considerably advanced (Beta et al., 2020).
- Analysis of pattern-forming mechanisms in reaction-diffusion systems traditionally relies on large, non-deforming, and isotropic domains, typically employing periodic or no-flux boundary conditions. But, for motile cells with continuously changing shapes, the boundaries bilaterally interact with the reaction-diffusion dynamics in the interior in known and unknown ways. While progress has been made in computations of such problems (see section *‘*Membrane confinement, deformations, and mechano-chemical feedbacks’), more mathematical theory is needed to fully understand the coupling between domain shape and internal reaction-diffusion dynamics, even more so in the presence of anisotropy (directional forces, such as externally applied electric fields [Yang et al., 2022], or coupling to microtubules [Dogterom and Koenderink, 2019]).

We hope that the survey provided here will stimulate future cross-disciplinary work to address these challenges and advance the field of intracellular pattern formation with its many biological and medical applications.
